# Utilizing Large language models to select literature for meta-analysis shows workload reduction while maintaining a similar recall level as manual curation

**DOI:** 10.1186/s12874-025-02569-3

**Published:** 2025-04-28

**Authors:** Xiangming Cai, Yuanming Geng, Yiming Du, Bart Westerman, Duolao Wang, Chiyuan Ma, Juan J. Garcia Vallejo

**Affiliations:** 1 Department of Molecular Cell Biology & Immunology, Amsterdam Infection & Immunity Institute and Cancer Center Amsterdam, Amsterdam UMC, Vrije Universiteit Amsterdam, Amsterdam, The Netherlands; 2 Department of Neurosurgery, Jinling Hospital, Nanjing, China; 3 Department of Neurosurgery, Affiliated Jingling Hospital, Nanjing Medical University, Nanjing, China; 4 Department of System Engineering and Engineering Management, The Chinese University of Hong Kong, Hong Kong, China; 5 Department of Neurosurgery, Cancer Center Amsterdam, Brain Tumor Center Amsterdam, Amsterdam UMC Location Vrije Universiteit Amsterdam, Amsterdam, The Netherlands; 6 Department of Clinical Sciences, Liverpool School of Tropical Medicine, Liverpool, UK; 7 School of Medicine, Southeast University, Nanjing, China; 8 Department of Neurosurgery, Affiliated Jinling Hospital, Medical School of Nanjing University, Nanjing, China; 9 Department of Neurosurgery, Jinling Hospital, the First School of Clinical Medicine, Southern Medical University, Nanjing, China

**Keywords:** Large language model, Meta-analysis, ChatGPT, Deepseek, Phi

## Abstract

**Background:**

Large language models (LLMs) like ChatGPT showed great potential in aiding medical research. A heavy workload in filtering records is needed during the research process of evidence-based medicine, especially meta-analysis. However, few studies tried to use LLMs to help screen records in meta-analysis.

**Objective:**

In this research, we aimed to explore the possibility of incorporating multiple LLMs to facilitate the screening step based on the title and abstract of records during meta-analysis.

**Methods:**

Various LLMs were evaluated, which includes GPT-3.5, GPT-4, Deepseek-R1-Distill, Qwen-2.5, Phi-4, Llama-3.1, Gemma-2 and Claude-2. To assess our strategy, we selected three meta-analyses from the literature, together with a glioma meta-analysis embedded in the study, as additional validation. For the automatic selection of records from curated meta-analyses, a four-step strategy called LARS-GPT was developed, consisting of (1) criteria selection and single-prompt (prompt with one criterion) creation, (2) best combination identification, (3) combined-prompt (prompt with one or more criteria) creation, and (4) request sending and answer summary. Recall, workload reduction, precision, and F1 score were calculated to assess the performance of LARS-GPT.

**Results:**

A variable performance was found between different single-prompts, with a mean recall of 0.800. Based on these single-prompts, we were able to find combinations with better performance than the pre-set threshold. Finally, with a best combination of criteria identified, LARS-GPT showed a 40.1% workload reduction on average with a recall greater than 0.9.

**Conclusions:**

We show here the groundbreaking finding that automatic selection of literature for meta-analysis is possible with LLMs. We provide it here as a pipeline, LARS-GPT, which showed a great workload reduction while maintaining a pre-set recall.

**Supplementary Information:**

The online version contains supplementary material available at 10.1186/s12874-025-02569-3.

## Introduction

The medical understanding of diseases has advanced rapidly during the last decades, but the translation from bench to bedside is lagging [1]. Evidence-based medicine (EBM), especially meta-analysis, facilitates the application of novel therapies into clinics; however, the processes of conducting meta-analysis are time-consuming and work intensive [2]. Artificial intelligence (AI) is becoming ubiquitous in medicine. [1] And AI-based solutions are developed to reduce human efforts spent on EBM with promising performance [3]. AI models can provide predicted probability for all records based on “similarity” between them. However, human annotators are needed to train the AI models [4, 5]. What’s more, although it helps to accelerate the research process, researchers still need to screen all records.

Recent releases of large language models (LLMs) like ChatGPT have dramatic implications on medical research; [6–8] however, few studies have evaluated its application in aiding EBM and review writing. Shaib et al. utilized ChatGPT (text-davinci- 003) to synthesize medical evidence, [9] and Shuai et al*.* explored its effectiveness in generating Boolean queries for a literature search [10]. However, almost no study has investigated its application in compensating or substituting human effort spent on filtering records during meta-analysis, a key issue because of the exponentially increased number of primary literature and systemic reviews required by medical researchers nowadays [11]. Kartchner et al. applied LLM to the extraction of clinical data from literature. However, they only tested the performance of GPT 3.5 Turbo and GPT-JT [12].

In this study, we aimed to explore the possibility of using LLM to aid the automatic selection of literature records (based on their title and abstract) for meta-analysis by developing a pipeline named LARS-GPT (Literature Records Screener based on ChatGPT-like LLM). With this study, we show a way to integrate LLMs into the field of EBM, which may impact the research pattern of meta-analysis.

## Methods

### Screen pipeline incorporating LLM: LARS-GPT

In general, the workflow of meta-analysis has the following steps: (1) define research question; (2) select literature databases and design search strategy; (3) screen records based on their titles and abstracts; (4) screen records based on full text of records; (5) extract and synthesize data. In the present study, we focused on incorporating LLM into the third step of this workflow.

**Fig. 1:**
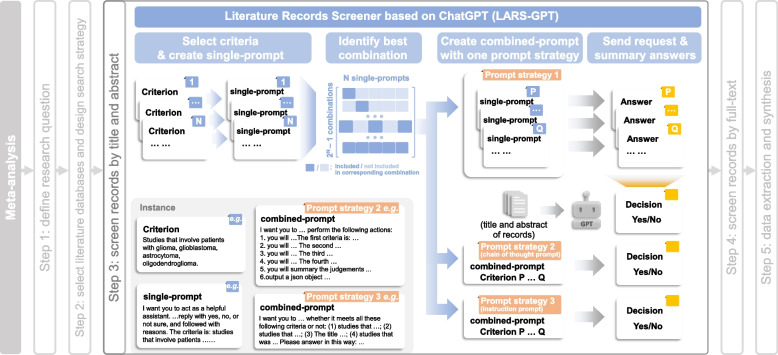
Schematic illustration of the LARS-GPT pipeline. Single-prompt represents a prompt with only one criterion. Combined-prompt stands for prompt with more than one criterion. Color of labels: single-prompt (blue), combined-prompt and prompt strategy (orange), and answer and decision (yellow)

To do that, we designed the four-step pipeline, LARS-GPT (Fig. 1). First, users need to select criteria (some suitable criteria from filtering criteria of meta-analysis) and create a prompt for each criterion (single-prompt; Table 1). Second, users need to evaluate these single-prompts using a few records and then select the best combination of single-prompts. Third, users need to choose a prompt strategy and merge single-prompts in the best combination to make a combined prompt (combined-prompt; Supplementary File 1) in accordance with the selected prompt strategy. Finally, the combined-prompt, together with the title and abstract of each record, will be submitted to LLM as chat completion. The decisions about whether a record meets the user’s criteria will then be extracted from returned answers. In practice, LARS-GPT could be performed in batches using Python.

**Table 1:** Representative prompt with single criterion (single-prompt)

single-prompt name	single-prompt content
Species	I want you to act as a helpful assistant. I will give you title and abstract of a publication and you will reply whether it meets our criteria or not. I want you to only reply with yes, no, or not sure, and followed with reasons. The criteria is: studies that use human as primary research subject
Disease	…. The criteria is: studies that involve patients with glioma, glioblastoma, astrocytoma, oligodendroglioma
Research type	…. The criteria is: studies that are prospective or retrospective cohort study, case–control study. Of note, these research types doesn't meet the criteria: cross-sectional study, randomized controlled trial, review, protocol or others
Age	…. The criteria is: studies that involve adult patients (at least 18 years old)
Protein related	…. The criteria is: The title and abstract must mention that the study is related to the consumption of protein (e.g., total dairy, milk, meat, fish, poultry, process meat, and egg)

### Models and parameter setting

In this study, we evaluated both GPT- 3.5 (gpt- 3.5-turbo- 0301) and GPT- 4 (gpt- 4–0314) using the API (Application Programming Interface) provided by OpenAI. We also evaluated Deepseek (DeepSeek R1 Distill (Qwen 7B)) [13], Qwen (Qwen2.5 7B) [14], Phi (phi- 4 14B) [15], Llama (Meta Llama 3.1 8B) [16], Gemma (Gemma 2 27B) [17] and Claude (Claude2-alpaca- 13B) [18]. LM Studio (version 0.3.10) is applied to download and access those LLMs locally. Temperature was set to be zero in LLMs, which means no randomness was introduced while generating answers.

### Selection of validation meta-analyses

To cover broad medical fields, we selected three high-quality published meta-analyses as validation datasets, which focused on inflammatory bowel diseases (IBD), [19] diabetes mellitus (DM), [20] and sarcopenia, [21] respectively (Table 2). These published meta-analyses provided clear search strategies for Medline/PubMed database and a complete list of records that remained after screening based on their titles and abstracts. Thanks to this, we were able to repeat their literature search in Medline/PubMed and match record list to obtain the correct answer that whether these identified records could pass the screening step in a real-world practice (Table 2; Supplementary File 2). On top of these published meta-analyses, we conducted a new meta-analysis about glioma. The protocol of the glioma meta-analysis was registered on PROSPERO (CRD42023425790). In doing so, we can evaluate the performance of LLM in a first-hand practice.

**Table 2:** Summary of meta-analyses included as validation datasets for LARS-GPT

First author	Field	Publication year	Journal	Original research			Our repetition (validation datasets)	
				**All identified records**	**Identified records from Medline/PubMed**	**Records preserved in title and abstract screen step**	**Identified records from Medline/PubMed**	**Records preserved in title and abstract screen step (Matched)**
Cai X	Glioma	NA	NA	8550	6020	272	1360	264
Talebi S	Inflammatory Bowel Diseases	2023	Adv Nutr	2755	1285	51	1284	45
Aune D	Diabetes Mellitus	2023	Eur J Epidemiol	5320	1040	216	1039	124
Beaudart C	Sarcopenia	2023	J Cachexia Sarcopenia Muscle	2293	NA	188	1293	122

The number of records used for each step evaluation is different, due to the requirements of each step, the workload, and the cost of money. In the final step evaluation with the combined-prompt, almost all records were used for the GPT- 3.5 evaluation. However, only 100 randomly selected records were used for the evaluation of other models, due to the limited funding and long generating time. The detailed randomization method used here can be found in Supplementary File 3.

#### Step1: Prompt strategy design

**Fig. 2:**
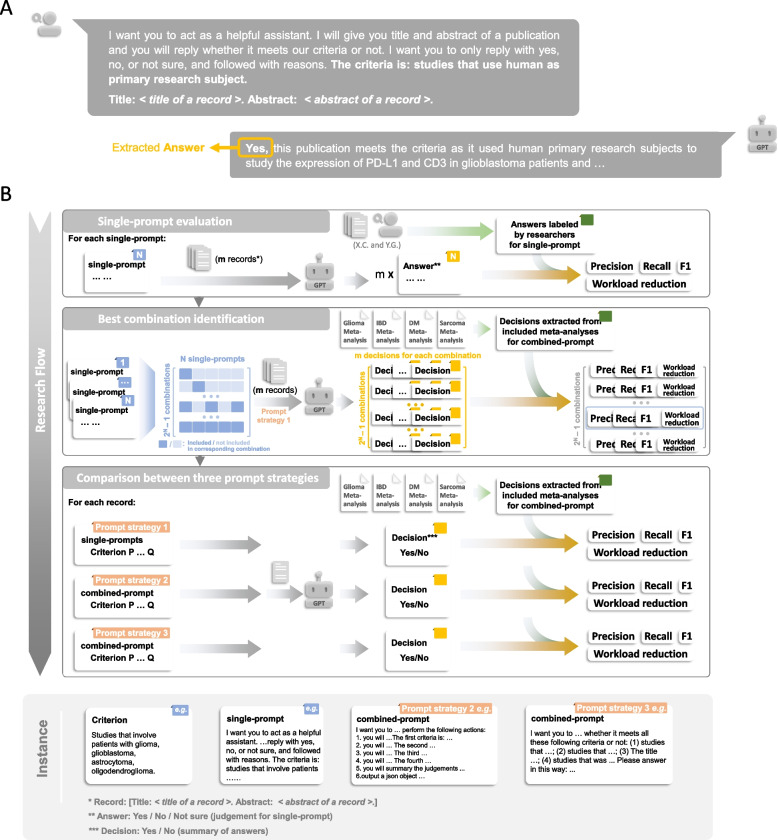
The research flow of this study. A representative case showing a request containing a single-prompt and the response from ChatGPT (**A**). The schematic illustrations of the research flow (**B**). Here also shows the detailed input (made by human researchers) for ChatGPT performance metrics calculation. Single-prompt represents a prompt with only one criterion. Combined-prompt stands for the prompt with more than one criterion. Color of labels: single-prompt (blue), combined-prompt and prompt strategy (orange), answer and decision (yellow), and true outcome of validation datasets (green)

We designed prompts (Table 1; Supplementary File 1) with the guidance from OpenAI (https://platform.openai.com/docs/guides/gpt-best-practices). However, the high flexibility of prompts and the “black box” nature of LLMs made it impossible to design a “best” prompt. In this study, we designed three distinct types of prompt strategies to help create better combined-prompt (Fig. 1 and 2; Supplementary File 1). For the “single criterion” prompt (prompt strategy 1), we simply maintain these single-prompts in the best combination. LLMs will respond to each single-prompt and determine whether a record meets each criterion or not. After receiving answers from LLMs, users need to summarize answers for each single-prompt and make a final decision for each record. In this study, as long as there is one answer that is “No”, the final decision for a record is “No”. Otherwise, the final decision will be “Yes”. For the “instruction prompt” (prompt strategy 3) and “chain of thought prompt” (prompt strategy 2), the best combination of single-prompts was merged into one combined-prompt (Fig. 1 and 2; Supplementary File 1). Users expect a final judgment from LLMs directly. In this research, we selected 4–5 criteria from each meta-analysis (Table 1; Supplementary File 1).

#### Step2: Evaluation of the classification performance of single-prompt

We (XC and YG) manually labeled the correct answers of each single-prompt within 100 randomly selected records (about 10 positive records and 90 negative records) for each validation meta-analysis. Here, records were called “positive records” if they remained after the screening step based on their titles and abstracts. Otherwise, they were called “negative records”. To avoid potential bias from the researchers, these records were manually labeled before we tested them on the LLMs. With these 100-reords datasets, we evaluated the performance of LLMs and a random classifier regarding single-prompts.

#### Step3: Evaluation of single-prompt combination and identification of best combination

Before conducting any evaluation, the “best” combination of single-prompts was unknown. In other words, how many single-prompts and which single-prompts should be selected for combined-prompt creation? To address this question, we evaluated all possible combinations of designed single-prompts. Among these combinations, we selected the best combination, which has a recall ≥ 0.9 and the best workload reduction.

### Statistical analysis

Because of the nature of ChatGPT, the generated answer from ChatGPT varies each time, even with exactly identical input. So, we assessed the robustness score of each single-prompt with repeated requests before testing the LARS-GPT pipeline (see Supplementary File 3). In general, the returns were stable, with a robustness score ranging from 0.747 to 0.996 (Supplementary Fig. 1 and 2). For other models, temperature was set to be zero to avoid introducing randomness.

The performance of LLMs was assessed with precision, recall, F1 score, and workload reduction metrics. The workload reduction indicator was defined as:

$${workload\;reduction=n}_{records\;excluded\;by\;model}/{n}_{all\;records}$$

where n is the number of records. The workload reduction indicator varies between 0 and 1, where 0 indicates none of the work was reduced and 1 signifies that all work was reduced. For meta-analysis, recall is the most significant indicator, followed with workload reduction, F1, and precision. Throughout the study, we placed greater emphasis on recall and workload reduction as the primary performance metrics.

For other machine learning (ML) models, it’s possible to reach a 100% recall with the compromise of low accuracy. However, due to the distinct mechanisms behind LLMs and other ML models, this is impossible for LLM-based solutions, at least for our LARS-GPT pipeline. So, in this study, a random classifier was used as a baseline reference (see Supplementary File 3). The classifications made by human researchers are used as “true decisions” to calculate the performance metrics of the LARS-GPT pipeline.

## Results

### Single-prompts exhibit distinct performance

The performance of each single-prompt was assessed (Table 3; Supplementary Table 1). Overall, the majority of prompts had better performance with LLMs than a random classifier. However, Claude- 2 had a precision similar to that of a random classifier. The mean recall for all LLM was 0.800, ranging from 0.72 for Phi- 4 to 0.89 for Gemma- 2. A total of 63.9% single-prompts had a recall higher than 0.8. Surprisingly, the recalls could be quite different between these two versions of GPT, even for the same single-prompt, *e.g.*, the “Control” single-prompt from sarcopenia meta-analysis (GPT- 3.5: 0.838; GPT- 4: 0.235; Supplementary Table 1) and the “Protein related” single-prompt from IBD meta-analysis (GPT- 3.5: 0.897; GPT- 4: 0.483; Supplementary Table 1).

**Table 3:** Performance of single-prompts from glioma meta-analysis using LLMs, and random classifier

**Glioma**					
**single-prompt**	**Model**	**Precision**	**Recall**	**F1**	**Workload reduction**
Species	GPT- 3.5	0.587	0.786	0.672	0.250
	GPT- 4	0.791	0.607	0.687	0.570
	Deepseek-R1-Distill	0.815	0.946	0.876	0.350
	Qwen- 2.5	0.840	0.750	0.792	0.500
	Phi- 4	0.878	0.643	0.742	0.590
	Llama- 3.1	0.700	0.750	0.724	0.400
	Gemma- 2	0.662	0.911	0.767	0.230
	Claude- 2	0.529	0.821	0.643	0.130
	Random classifier	0.557	0.494	0.523	0.504
Disease	GPT- 3.5	0.989	0.905	0.945	0.130
	GPT- 4	1.000	1.000	1.000	0.050
	Deepseek-R1-Distill	1.000	0.705	0.827	0.330
	Qwen- 2.5	1.000	0.979	0.989	0.070
	Phi- 4	1.000	0.979	0.989	0.070
	Llama- 3.1	1.000	0.937	0.967	0.110
	Gemma- 2	1.000	0.979	0.989	0.070
	Claude- 2	0.958	0.716	0.820	0.290
	Random classifier	0.950	0.506	0.659	0.494
Treatment	GPT- 3.5	0.530	0.917	0.672	0.170
	GPT- 4	0.745	0.792	0.768	0.490
	Deepseek-R1-Distill	0.721	0.646	0.681	0.570
	Qwen- 2.5	0.764	0.875	0.816	0.450
	Phi- 4	0.917	0.688	0.786	0.640
	Llama- 3.1	0.955	0.438	0.601	0.780
	Gemma- 2	0.655	0.792	0.717	0.420
	Claude- 2	0.472	0.875	0.613	0.110
	Random classifier	0.485	0.504	0.494	0.501
Research type	GPT- 3.5	0.915	0.966	0.940	0.060
	GPT- 4	0.946	0.989	0.967	0.070
	Deepseek-R1-Distill	0.929	0.584	0.717	0.440
	Qwen- 2.5	0.977	0.966	0.971	0.120
	Phi- 4	0.944	0.955	0.949	0.100
	Llama- 3.1	0.978	0.978	0.978	0.110
	Gemma- 2	0.978	0.978	0.978	0.110
	Claude- 2	0.939	0.697	0.800	0.340
	Random classifier	0.893	0.495	0.635	0.506

Different single-prompts also exhibited distinct recalls. Most single-prompts performed well, like the “Species” prompt from DM meta-analysis (all models > 0.8; Supplementary Table 1). However, few single-prompts demonstrated low recalls, *e.g.*, the “Control” prompt from Sarcopenia meta-analysis (Qwen- 2.5: 0.191; Llama- 3.1: 0.044; GPT- 4:0.235; Supplementary Table 1).

### The best combination of single-prompts is identified by evaluating the performance of all possible combinations

All combinations of single-prompts were shown in the form of UpSet plots (Supplementary Fig. 3–6). As expected, when the number of single-prompts increases, the recall tends to decrease, while workload reduction and precision increase. In general, most combinations presented superior performance compared to a random classifier. To our surprise, it’s not uncommon to find a combination with three single-prompts having a recall of 0.9 or higher.

Based on the preset threshold, we identified the best combination with the highest workload reduction from combinations that have a recall greater than 0.9. However, in some cases, there was only one combination with a recall ≥ 0.9, which only included one single-prompt. Because we wanted to evaluate the performance of prompt strategies 2 and 3, which were specifically tailored for combinations involving multiple single-prompts, we selected another combination instead as a sub-best combination for the following analyses.

### Three prompt strategies show similar performance

Full combination (including all designed single-prompts) and best combination were both evaluated with three prompt strategies (Supplementary Table 2; Supplementary File 1). Obviously, the best combinations had ideal and much better recalls than full combinations (mean recalls: 0.876 vs. 0.540) and random classifier. The best combinations demonstrated remarkable recalls ranged from 0.889 to 1.000 in 65.5% cases. The corresponding workload reductions varied from 0 to 0.890, with an average of 0.401.

**Fig. 3:**
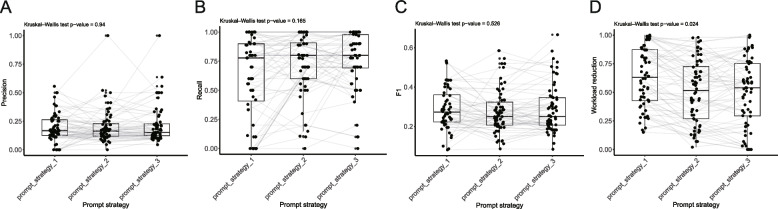
Comparison of the performance of best and full combinations between three prompt strategies. Comparison of the performance between three prompt strategies, regarding precision (**A**), recall (**B**), F1 score (**C**), and workload reduction (**D**)

**Fig. 4:**
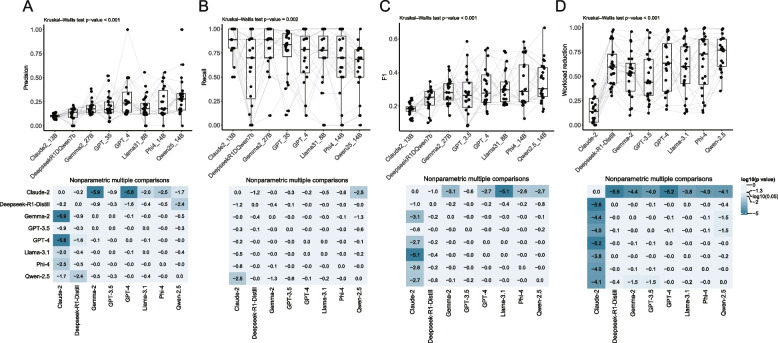
Comparison of the performance of best and full combinations between LLMs. Comparison of the performance between 8 models, regarding precision (**A**), recall (**B**), F1 score (**C**), and workload reduction (**D**). The lower panel shows the results of the corresponding non-parametric multiple comparison with a log10 transformed p value

These three prompt strategies showed comparable levels of performance (Fig. 3), regarding all four metrics. However, these 8 included models had distinct performance (Fig. 4). Claude- 2 showed statistically lower precision and F1 scores compared with Gemma- 2, GPT- 4, Limma- 3.1, Phi- 4, and Qwen- 2.5 (Fig. 4A and 4 C). The recall is similar between models, except that Claude- 2 had a higher recall than Qwen- 2.5 (Fig. 4B). Also, Claude- 2 showed a much lower workload reduction than other models (Fig. 4D).

## Discussion

In this research, we developed LARS-GPT and proved that it can greatly reduce the filtering workload while maintaining an ideal recall during the screening step based on the titles and abstracts of records for meta-analysis.

The mechanism employed by LLMs is different from that of previous AI models. Previous AI models applied active learning to select the training dataset and returned all records ordered by a “similarity” index [5]. However, LLMs have been trained to predict text that follows the input text. By doing so, LLMs can directly answer questions and return whether an input record meets the provided criteria or not. Due to the distinct mechanisms applied, previous AI models can reach a perfect recall with the compromise of low accuracy, but not for LLM-based methods. Thus, we excluded previous AI models as baseline references for performance evaluation in this study.

LLMs have advantages over previous AI models. One advantage is that extra training is unnecessary when applying LLMs to a new meta-analysis (although fine-tuning is possible) because LLMs were pre-trained on large-scale datasets. In comparison, a training dataset is required for every new meta-analysis if choosing previous AI models. Additionally, users do not need to worry about the imbalanced data problem [5] when using LARS-GPT for the same reason.

An obvious benefit of LARS-GPT is that it could be easily adapted to other LLMs by simply changing the API, since most LLMs work similarly. However, the performance of LARS-GPT depends on the performance of the LLM used, which is not guaranteed. We also believe that a well-performed prompt could be used for other LLMs. However, further research is needed to verify this idea of adapting LARS-GPT to other LLMs.

LLM hallucinations are one issue that has been emphasized in research. These hallucinations occur when a LLM makes up fake information and describes it like it is real [22, 23]. LARS-GPT avoids this issue because users need to provide the titles and abstracts of records to ChatGPT, rather than having ChatGPT search for the information. Nonetheless, we did observe instances where ChatGPT made false causal inferences. For example, ChatGPT might give a reason supporting a record meeting one filtering criterion, which is then followed by an opposite judgment. A similar false conclusion may occur when users ask ChatGPT to summarize a final judgment, *e.g.,* “The publication meets criterion 1, but not criterion 2. So, the publication meets all your criteria.” Despite occasional false judgment, LARS-GPT demonstrated an ideal performance in the current research.

Surprisingly, in this study, GPT- 4’s performance was not much better than GPT- 3.5. Although GPT- 4 may be more accurate, it could have lower recall compared to GPT- 3.5 (Supplementary Table 2), and recall is much more important than precision when screening literature for a meta-analysis. Furthermore, when evaluating the performance of three prompt strategies, Claude- 2 showed lower precision, F1 score, and workload reduction than other models (Fig. 4). The other 7 models had similar performance across all measures. In short, in the context of this research, no model was overwhelmingly superior to the other one, except Claude- 2.

It is important to evaluate the performance of LARS-GPT in various scenarios. Thus, in the study, we selected 4 meta-analyses with distinct types of diseases, which stand for cancer, immune-related disease, metabolic-related disease, and skeletal muscle disorder, respectively. In general, LARS-GPT demonstrated an ideal performance on all of them (Supplementary Table 2). What really impacts the performance of LARS-GPT is the prompts designed, which also highlights the value of prompt design steps in our pipeline.

In this study, a single-prompt is developed from a single filtering criteria, and a key step of single-prompt creation is the selection of criteria. Potential criteria should be derived from the inclusion and exclusion criteria of the designed meta-analysis. In some cases, however, researchers need to extract information from a subgroup analysis, which may not be presented in the title and abstract of a record, *e.g.* materials used in surgery, [24] and criteria related to such information are not suitable for prompt creation. To avoid this issue, it is better to use options that are more likely to be adequately judged using only the title and abstract of the record, which are criteria related to “Species”, “Disease”, and “Research type”. In fact, the majority of the best combinations identified in the current research were based on these three criteria. Thus, users are recommended to try them first when using LARS-GPT.

To apply LARS-GPT, users need to manually label a few records for single-prompts so that the best combination can be identified. Based on our experiences, to be well evaluated, each single-prompt needs around 10 positive and 10 negative records. Considering overlaps between the records for single-prompts, researchers need to label about 20 to 100 records for five single-prompts. Once an application based on LARS-GPT is developed, it will be much easier to do this labeling.

We tried three prompt strategies, including a “chain of thought prompt” (prompt strategy 2) that was designed following the OpenAI's guidelines. Surprisingly, all three prompt strategies showed comparable performance (Supplementary Table 2; Fig. 3). Indeed, the “chain of thought prompt” takes more time for LLMs to answer in a more organized format. However, this improvement does not translate into enhanced performance in LARS-GPT. A possible reason is that the two other “less structured” strategies already guided LLMs sufficiently. However, due to the “black box” nature of LLMs, we cannot explain the phenomenon. As a result, users are recommended to select whichever they prefer.

In our research, we did not use metrics like Work Saved over Sampling (WSS) and Average Time to Discover (ATD), [5] which have been commonly used to evaluate previous AI models. This is because LARS-GPT works in a completely different way, as mentioned before. Within LARS-GPT, LLMs will directly answer whether to include or exclude a record, instead of returning a probability for it.

In the filtering step of meta-analysis, a high recall is of very priority. There is a possibility that recalls are not satisfied, even though we have included a “recall > 0.9” criteria in choosing the best combination in the LARS-GPT. The balance between recall and precision is always a difficult issue to be addressed. Users might try some more single criteria in the beginning of LARS-GPT to have a best combination with high recall. Also, it is worthwhile to randomly check the filtered results after applying LLM.

## Conclusion

This study developed a pipeline named LARS-GPT, and using this pipeline showed that an automatic selection of records for a meta-analysis is possible with LLMs. Three prompt strategies showed similar performance. All LLMs evaluated, except Claude- 2, also have comparable performance. Further research may incorporate LLMs (and the multiple LLMs approach) into other steps of the meta-analysis workflow.

## Supplementary Information

Supplementary Material 1.

Supplementary Material 2.

## Data Availability

The original code used in this paper is available on GitHub (https://github.com/xiangmingcai/LARS). All responses from LLMs can be found in Supplementary File 2. Any additional information required is available from the corresponding author upon request.
