# Supplementary material for: Utilizing Large language models to select literature for meta-analysis shows workload reduction while maintaining a similar recall level as manual curation
Source: BMC Med Res Methodol. 2025 Apr 28;25:116. doi: 10.1186/s12874-025-02569-3 (PMC12036192; doi:10.1186/s12874-025-02569-3)
Supplement: Supplementary file 1 — Supplementary Material 1. [file 12874_2025_2569_MOESM1_ESM.pdf]

Supplementary Figures 2

Supplementary Figure 1 The responses of GPT-3.5 show high robustness in classifying records 2

Supplementary Figure 2 The other returns for robustness evaluation 3

Supplementary Figure 3 Performance of all combinations of single-prompts from glioma meta-analysis 6

Supplementary Figure 4 Performance of all combinations of single-prompts from inflammatory bowel diseases meta-analysis 9

Supplementary Figure 5 Performance of all combinations of single-prompts from diabetes mellitus meta-analysis 12

Supplementary Figure 6 Performance of all combinations of single-prompts from sarcopenia meta-analysis 15

Supplementary Tables 16

Supplementary Table 1 Performance of single-prompts from inflammatory bowel diseases, diabetes mellitus, and sarcopenia meta-analyses using LLMs, and random classifier 16

Supplementary Table 2 Performance of three prompt strategies with the best combination and the full combination 19

Supplementary Files 22

Supplementary File 1 The content of three prompt strategies using full combination 22

Supplementary File 2 Validation datasets from four meta-analyses 30

Supplementary File 3 Supplementary Methods 31

Supplementary Figures

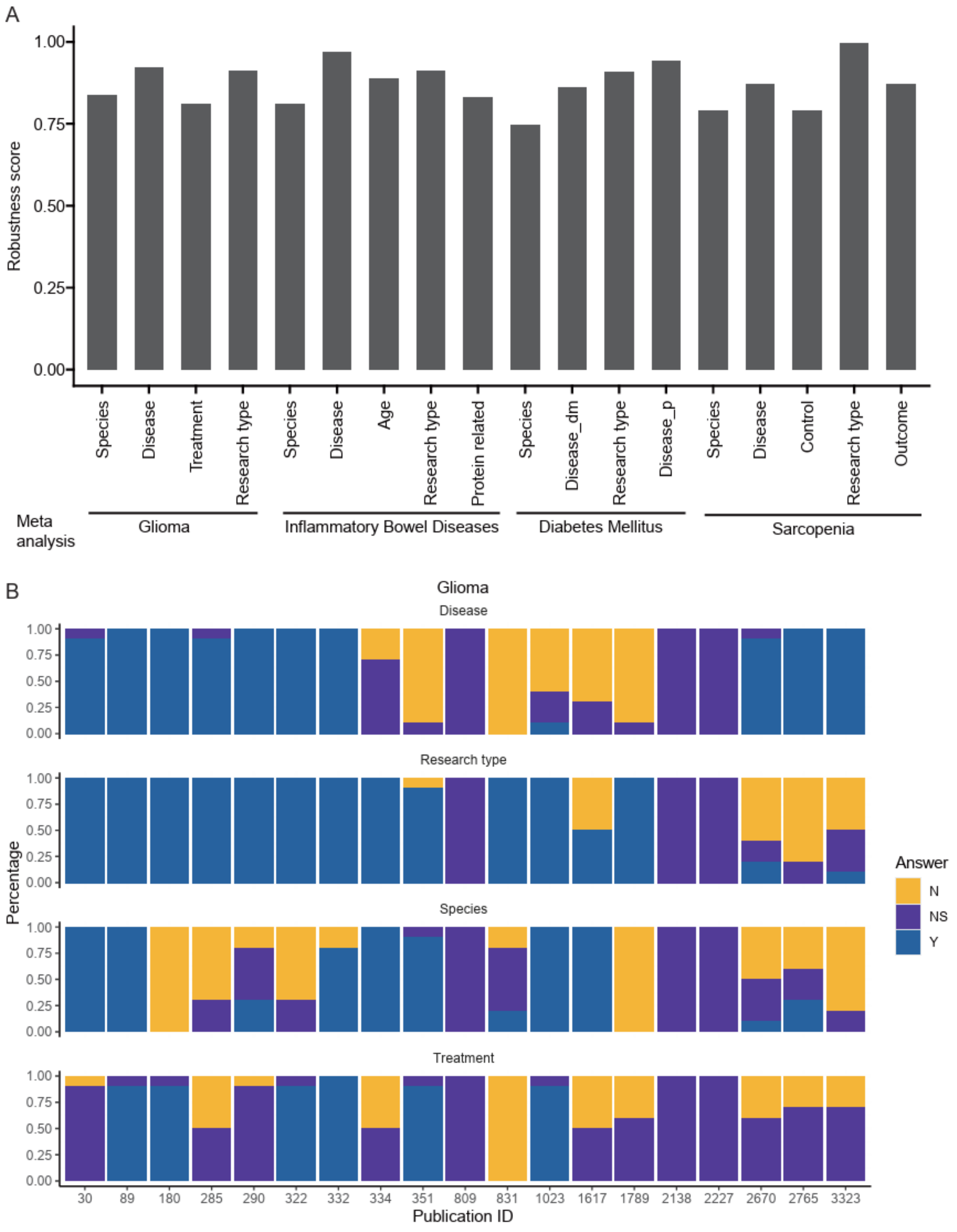

Supplementary Figure 1 The responses of GPT-3.5 show high robustness in classifying records

(**A**) The bar plot shows the robustness score of each single-prompt from the four meta-analyses included. (**B**) The stack bar plot shows the answers of repeated requests sent to GPT-3.5 with single-prompts from glioma meta-analysis. N, no; Y, yes; NS, not sure.

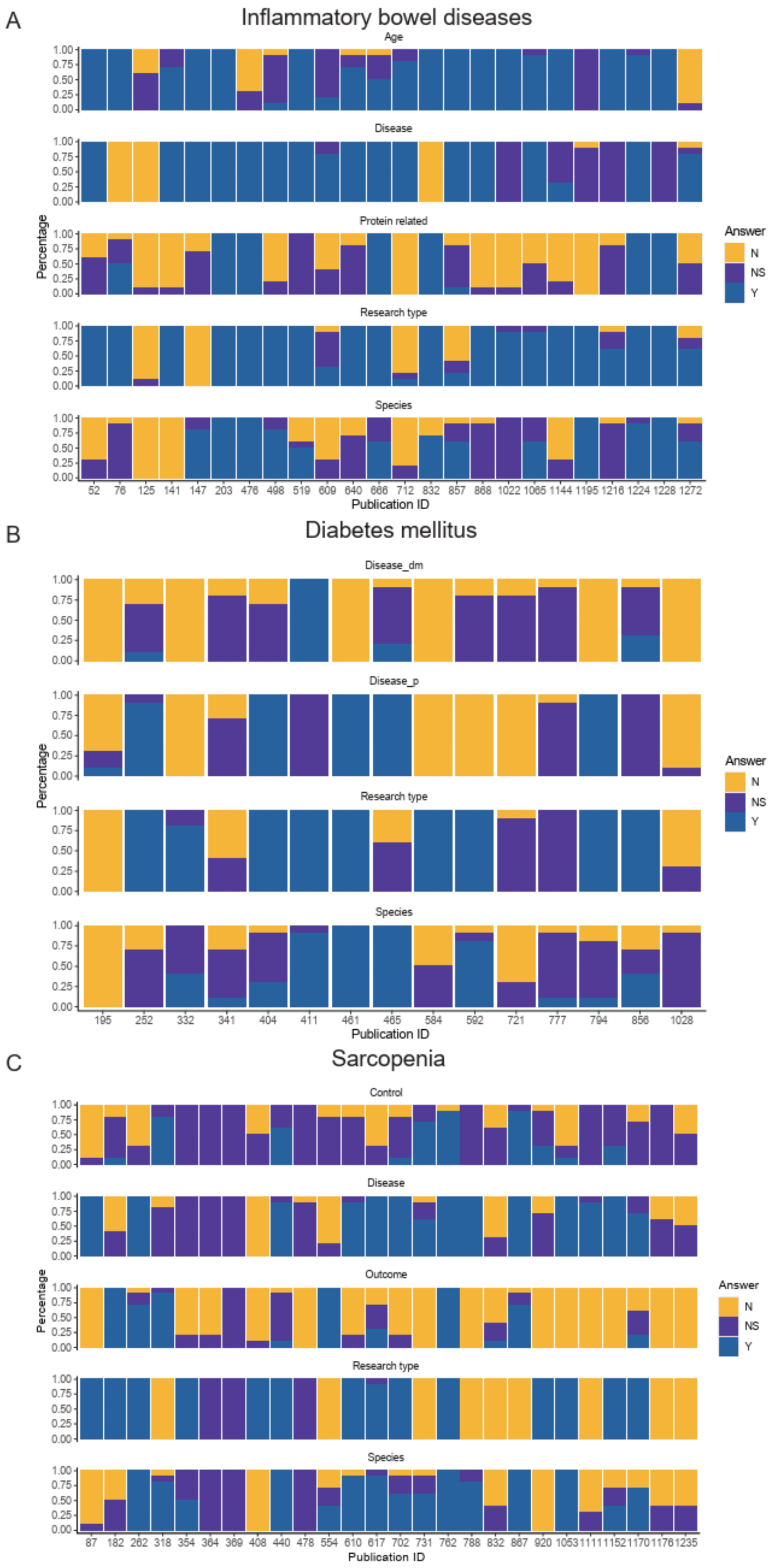

Supplementary Figure 2 The other returns for robustness evaluation

The stack bar plots show the answers of repeated requests sent to GPT-3.5 with single-prompts from inflammatory bowel diseases (**A**), diabetes mellitus (**B**), and sarcopenia (**C**) meta-analyses, respectively. N, no; Y, yes; NS, not sure.

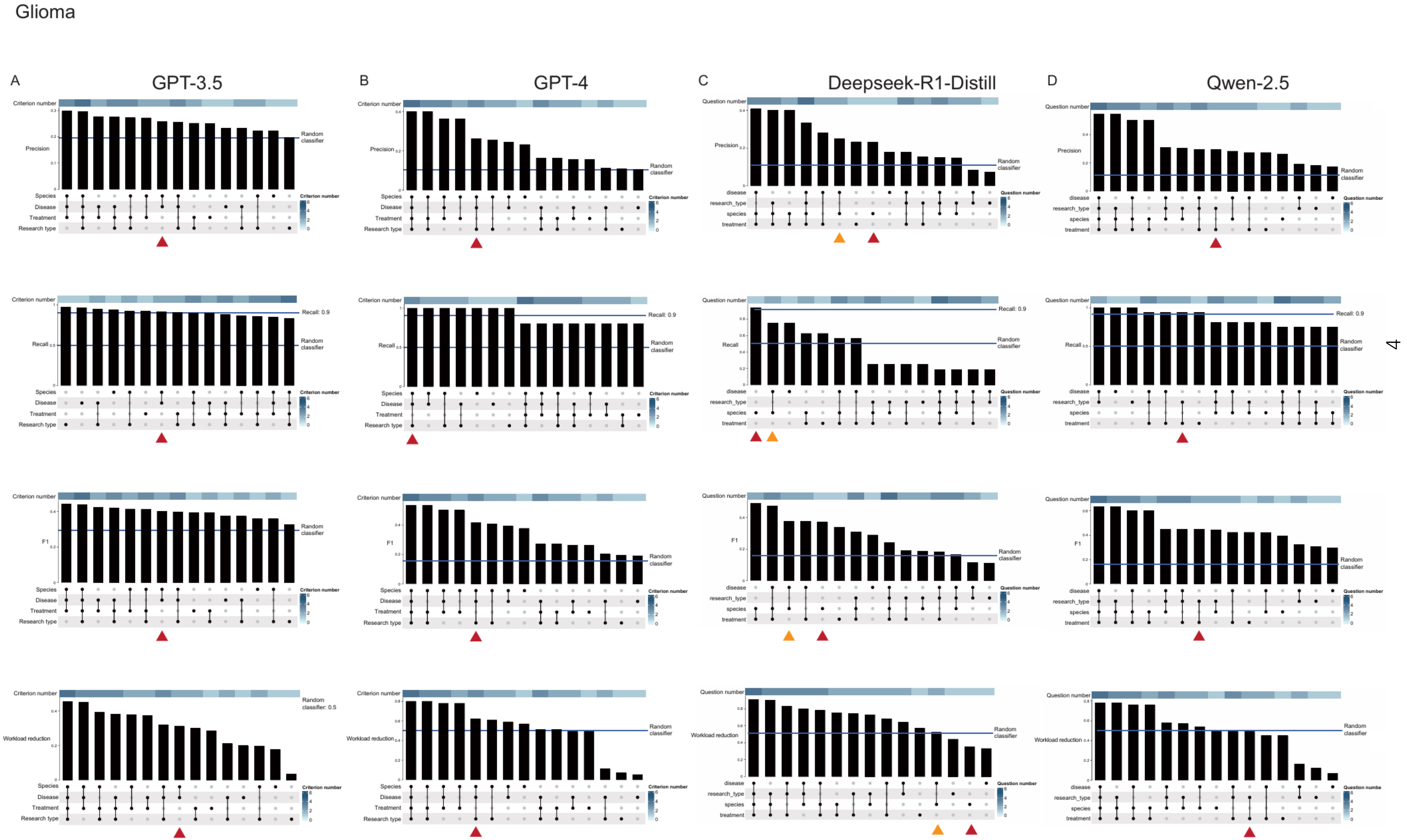
Glioma
A
GPT-3.5
B
GPT-4
C
Deepseek-R1-Distill
D
Qwen-2.5
Criterion number
Question number
Precision
Recall
F1
Workload reduction
Random classifier
Random classifier: 0.5
Recall: 0.9
Species
Disease
Treatment
Research type
disease
research_type
species
treatment

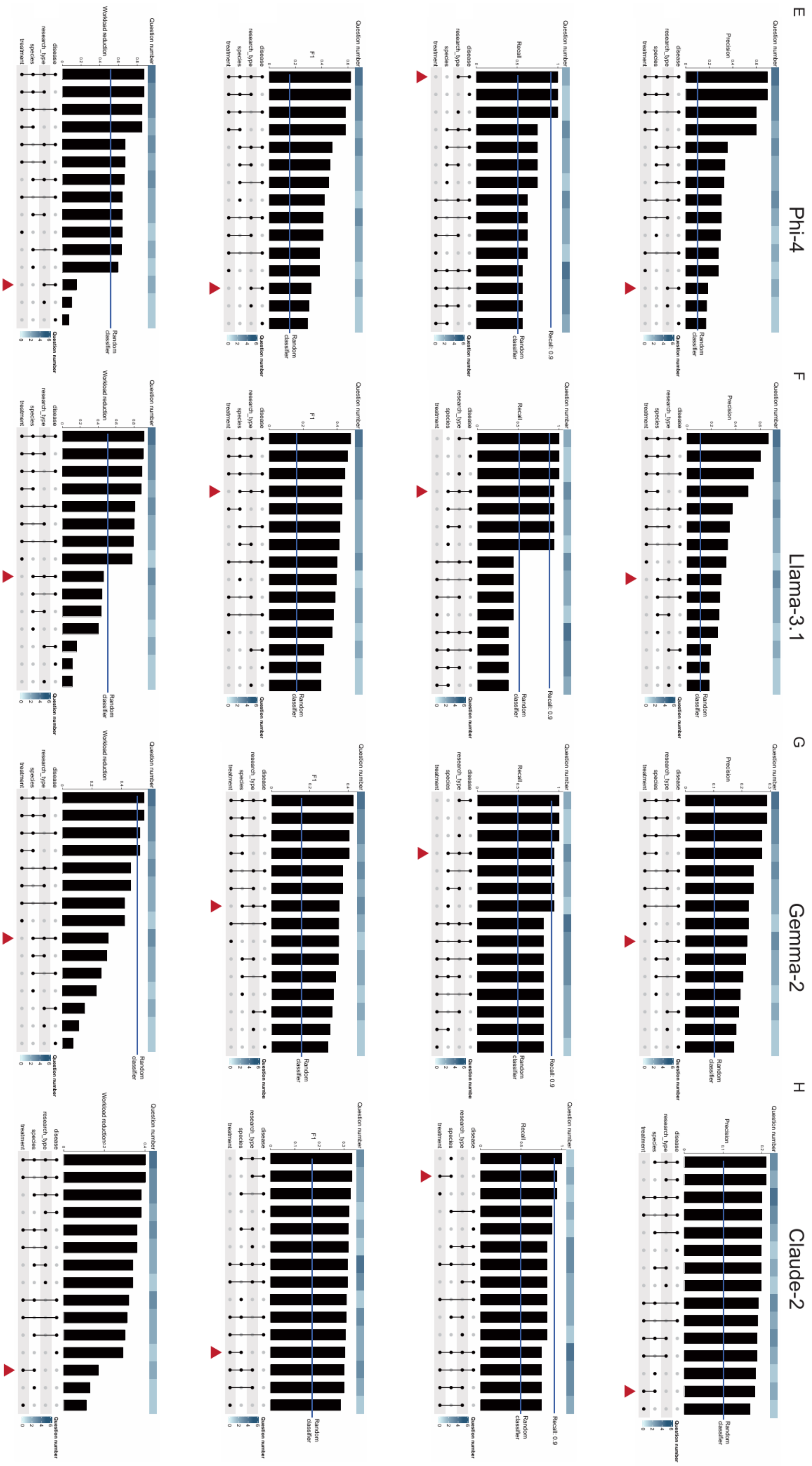
E
F
G
H
Phi-4
Llama-3.1
Gemma-2
Claude-2
Question number
Precision
Recall
F1
Workload reduction
Random classifier
Recall: 0.9
disease
research_type
species
treatment

Supplementary Figure 3 Performance of all combinations of single-prompts from glioma meta-analysis

In the Upset plot, the bar chart above represents the evaluation metrics. The dotted line at the bottom presents the single-prompts included in the corresponding combination. Precision, recall, F1 score, and workload reduction are presented in each sub figure separately. for GPT-3.5 (**A**), GPT-4 (**B**), Deepseek-R1-Distill (**C**), Qwen-2.5 (**D**), Phi-4 (**E**), Llama-3.1 (**F**), Gemma-2 (**G**), and Claude-2 (**H**) are presented respectively. Best combination is marked with a triangle. Sub-best combination is marked with a yellow triangle.

Inflammatory bowel diseases

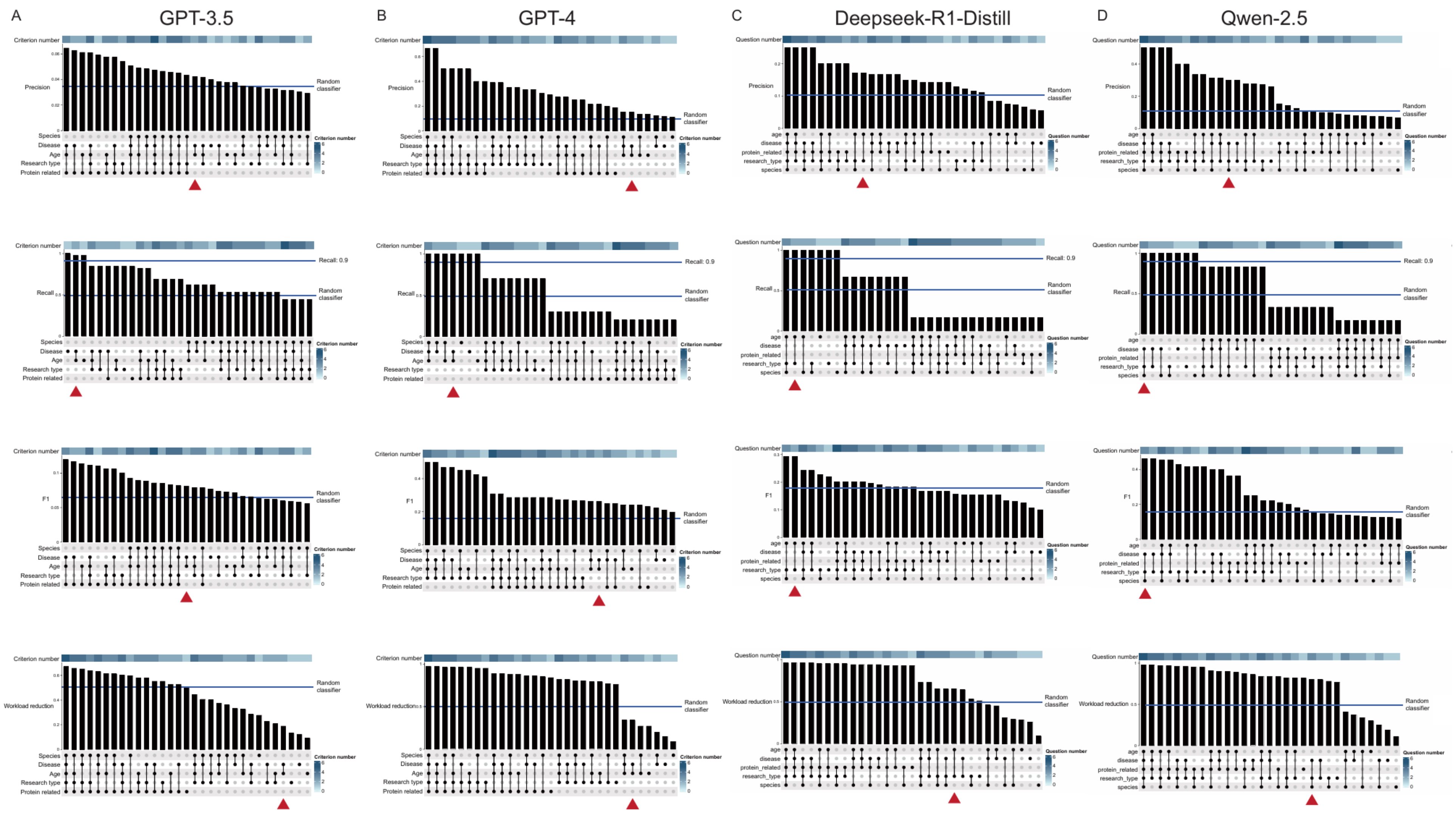

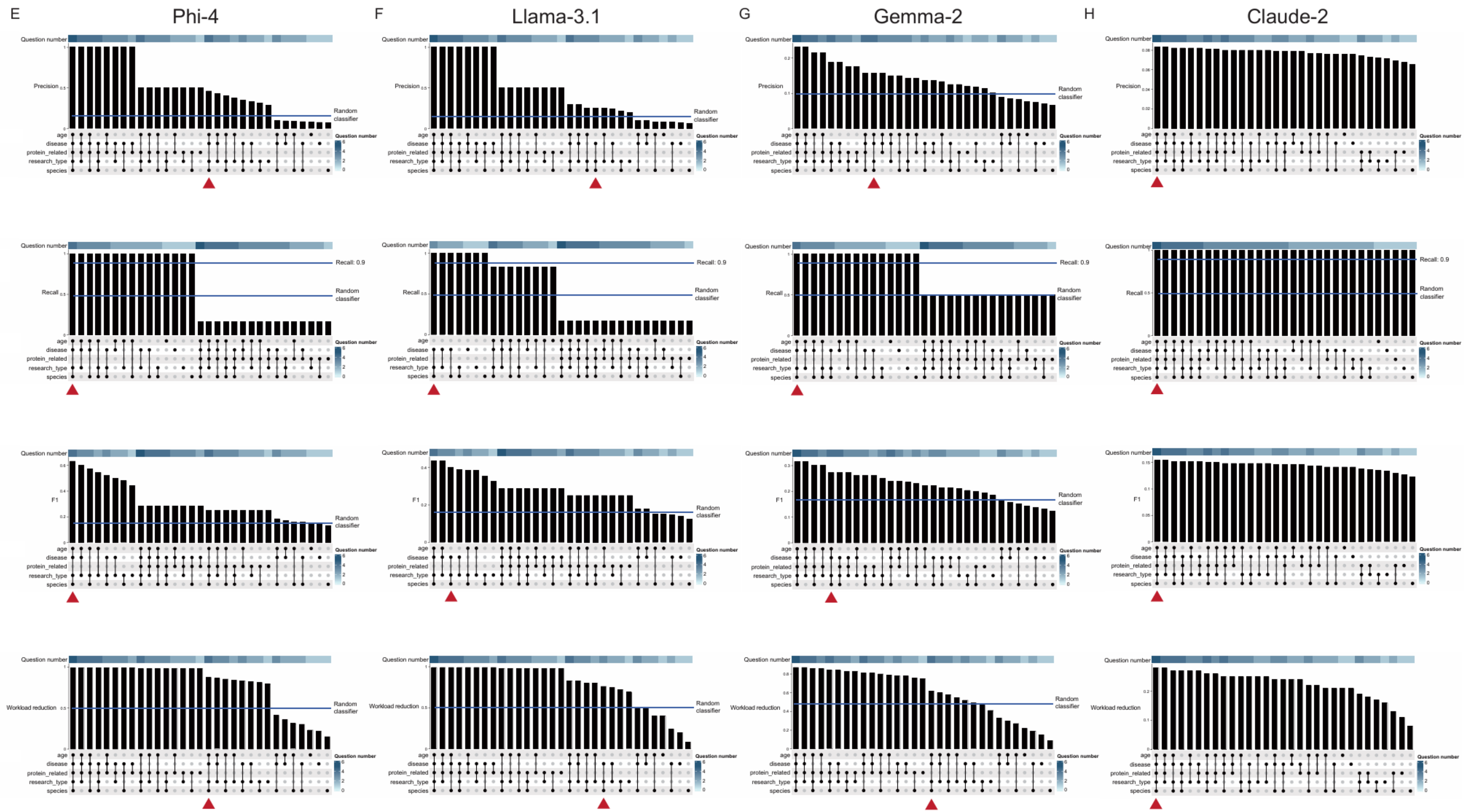
E
Phi-4
F
Llama-3.1
G
Gemma-2
H
Claude-2
Question number
Precision
Recall
Recall: 0.9
F1
Workload reduction
Random classifier
age
disease
protein_related
research_type
species

Supplementary Figure 4 Performance of all combinations of single-prompts from inflammatory bowel diseases meta-analysis

In the Upset plot, the bar chart above represents the evaluation metrics. The dotted line at the bottom presents the single-prompts included in the corresponding combination. Precision, recall, F1 score, and workload reduction are presented in each sub figure separately. for GPT-3.5 (**A**), GPT-4 (**B**), Deepseek-R1-Distill (**C**), Qwen-2.5 (**D**), Phi-4 (**E**), Llama-3.1 (**F**), Gemma-2 (**G**), and Claude-2 (**H**) are presented respectively. Best combination is marked with a triangle.

Diabetes mellitus

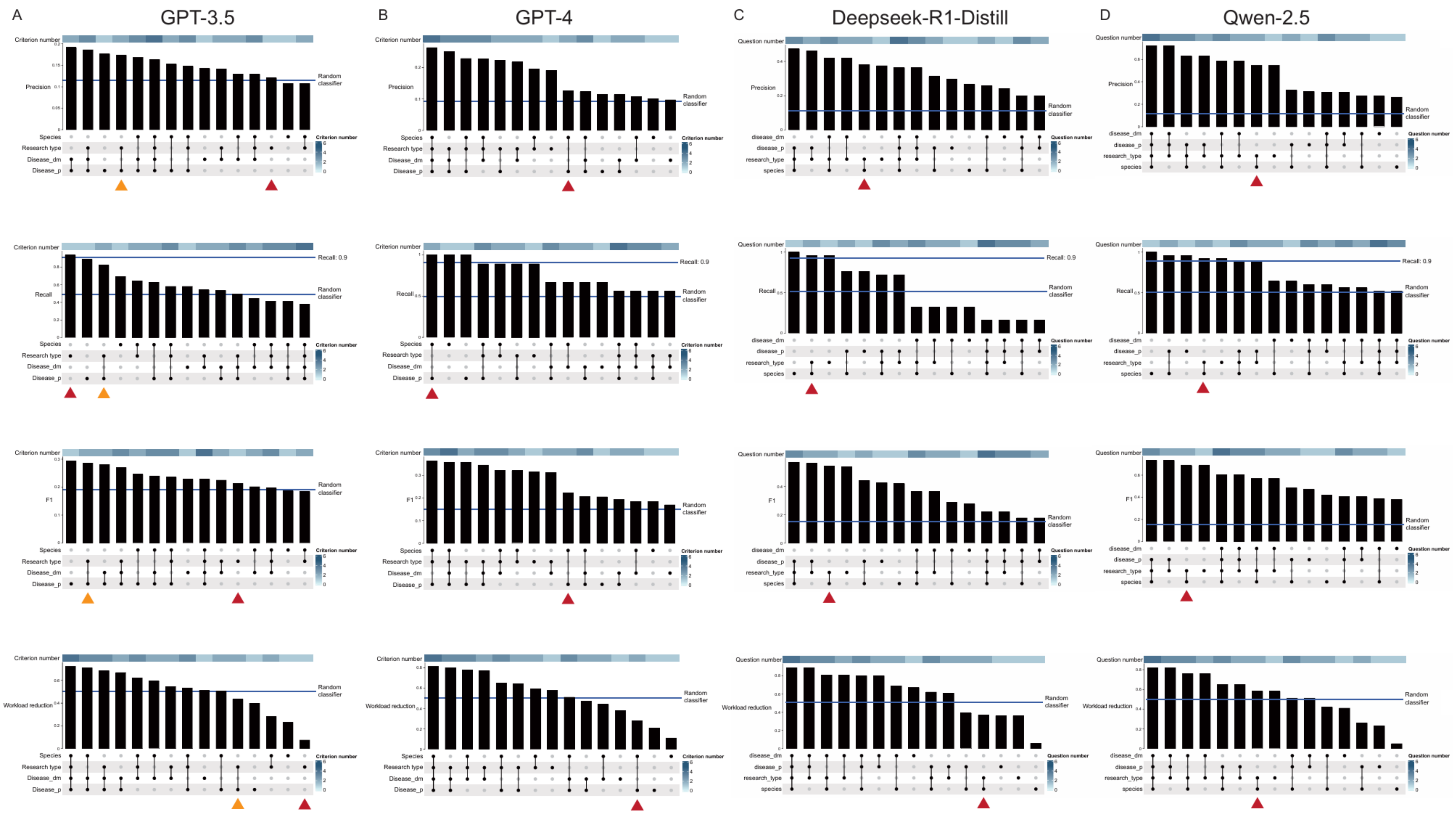

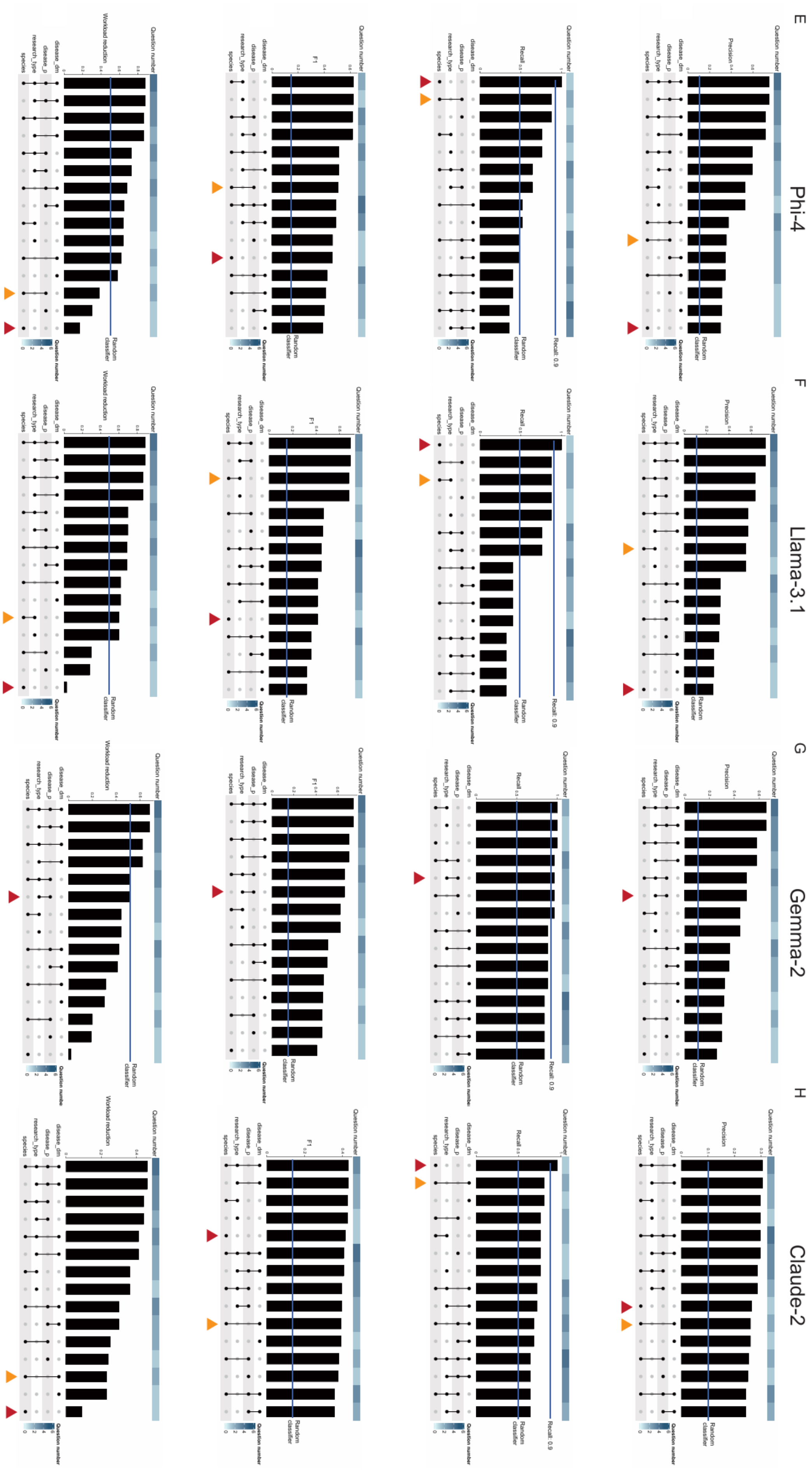
E
Phi-4
F
Llama-3.1
G
Gemma-2
H
Claude-2
Question number
Precision
Recall
F1
Workload reduction
Random classifier
Recall: 0.9
disease_dm
disease_p
research_type
species

Supplementary Figure 5 Performance of all combinations of single-prompts from diabetes mellitus meta-analysis

In the Upset plot, the bar chart above represents the evaluation metrics. The dotted line at the bottom presents the single-prompts included in the corresponding combination. Precision, recall, F1 score, and workload reduction are presented in each sub figure separately. for GPT-3.5 (**A**), GPT-4 (**B**), Deepseek-R1-Distill (**C**), Qwen-2.5 (**D**), Phi-4 (**E**), Llama-3.1 (**F**), Gemma-2 (**G**), and Claude-2 (**H**) are presented respectively. Best combination is marked with a triangle. Sub-best combination is marked with a yellow triangle.

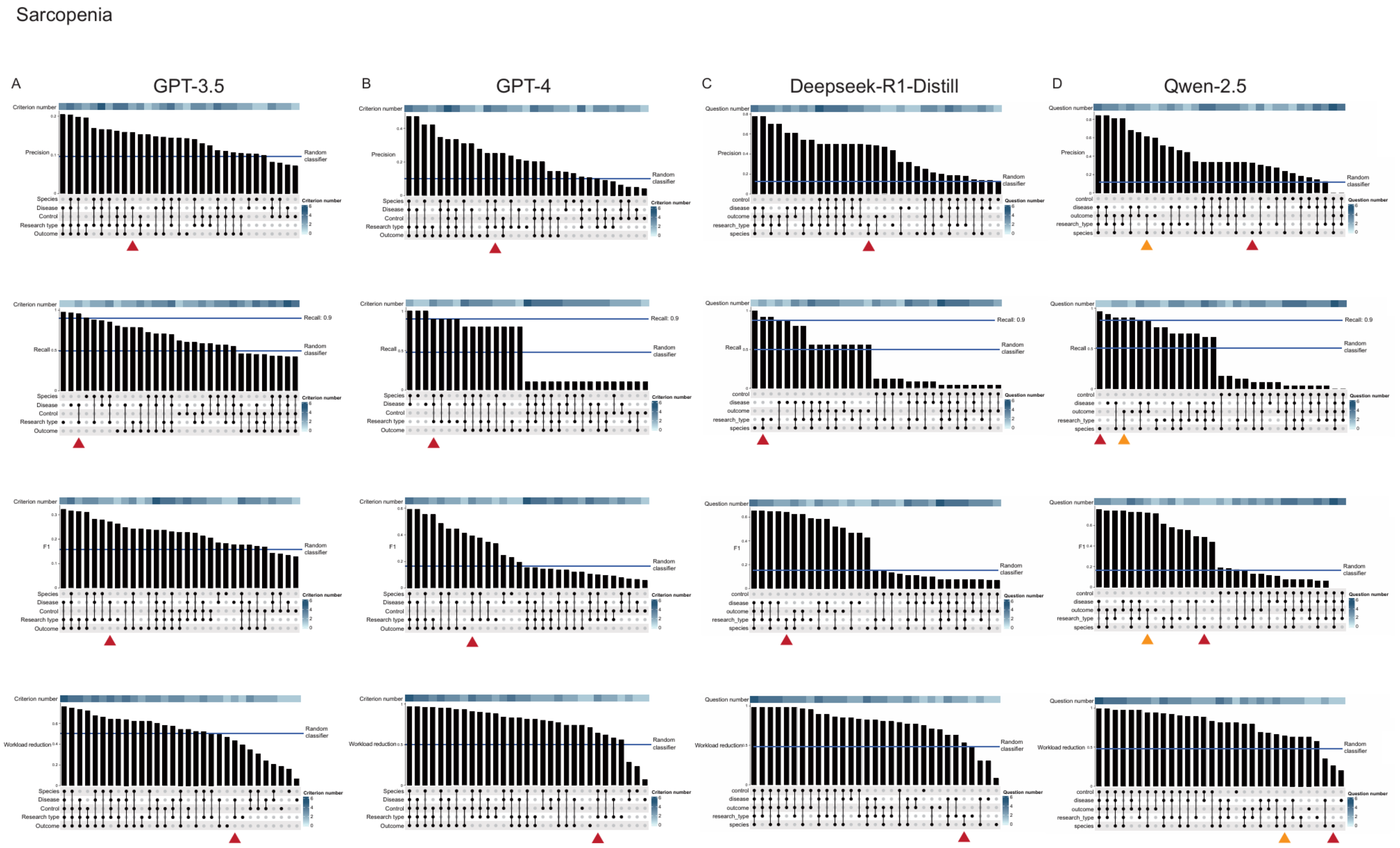
Sarcopenia
A
GPT-3.5
B
GPT-4
C
Deepseek-R1-Distill
D
Qwen-2.5
Criterion number
Question number
Precision
Recall
F1
Workload reduction
Random classifier
Recall: 0.9
Species
Disease
Control
Research type
Outcome
control
disease
outcome
research_type
species

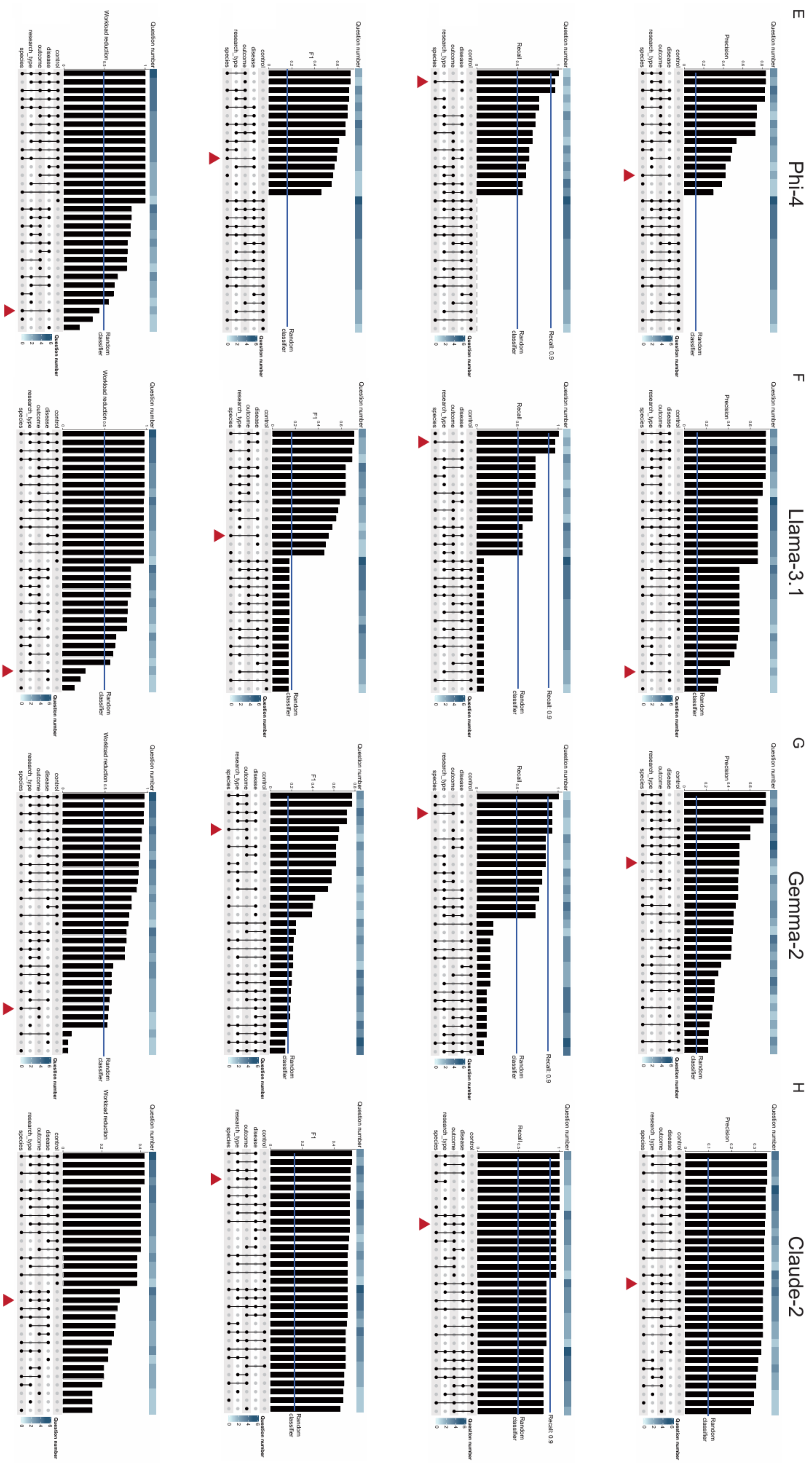
E
Phi-4
F
Llama-3.1
G
Gemma-2
H
Claude-2
Question number
Precision
Recall
F1
Workload reduction
Random classifier
Recall: 0.9
control
disease
outcome
research_type
species

Supplementary Figure 6 Performance of all combinations of single-prompts from sarcopenia meta-analysis

In the Upset plot, the bar chart above represents the evaluation metrics. The dotted line at the bottom presents the single-prompts included in the corresponding combination. Precision, recall, F1 score, and workload reduction are presented in each sub figure separately. for GPT-3.5 (**A**), GPT-4 (**B**), Deepseek-R1-Distill (**C**), Qwen-2.5 (**D**), Phi-4 (**E**), Llama-3.1 (**F**), Gemma-2 (**G**), and Claude-2 (**H**) are presented respectively. Best combination is marked with a triangle. Sub-best combination is marked with a yellow triangle.

Supplementary Tables

Supplementary Table 1 Performance of single-prompts from inflammatory bowel diseases, diabetes mellitus, and sarcopenia meta-analyses using LLMs, and random classifier

Inflammatory Bowel Diseases

**single-prompt**	**Model**	**Precision**	**Recall**	**F1**	**Workload reduction**
Species	GPT-3.5	0.973	0.780	0.866	0.270
	GPT-4	0.989	0.989	0.989	0.090
	Deepseek-R1-Distill	0.989	0.989	0.989	0.090
	Qwen-2.5	0.989	0.967	0.978	0.110
	Phi-4	0.988	0.923	0.954	0.150
	Llama-3.1	0.978	0.989	0.983	0.080
	Gemma-2	1.000	1.000	1.000	0.090
	Claude-2	0.902	0.912	0.907	0.080
	Random classifier	0.909	0.496	0.641	0.504
Disease	GPT-3.5	0.911	0.965	0.937	0.100
	GPT-4	0.965	0.965	0.965	0.150
	Deepseek-R1-Distill	0.986	0.859	0.918	0.260
	Qwen-2.5	0.975	0.929	0.951	0.190
	Phi-4	0.987	0.906	0.945	0.220
	Llama-3.1	0.988	0.929	0.958	0.200
	Gemma-2	0.965	0.965	0.965	0.150
	Claude-2	0.861	0.800	0.829	0.210
	Random classifier	0.856	0.509	0.637	0.494
Research type	GPT-3.5	0.241	1.000	0.389	0.130
	GPT-4	0.750	0.857	0.800	0.760
	Deepseek-R1-Distill	0.388	0.905	0.543	0.510
	Qwen-2.5	0.696	0.762	0.728	0.770
	Phi-4	0.714	0.714	0.714	0.790
	Llama-3.1	0.548	0.810	0.654	0.690
	Gemma-2	0.356	1.000	0.525	0.410
	Claude-2	0.238	0.952	0.381	0.160
	Random classifier	0.201	0.479	0.283	0.501
Age	GPT-3.5	0.819	1.000	0.901	0.060
	GPT-4	1.000	0.948	0.973	0.270
	Deepseek-R1-Distill	0.944	0.883	0.912	0.280
	Qwen-2.5	0.971	0.883	0.925	0.300
	Phi-4	0.935	0.935	0.935	0.230
	Llama-3.1	0.983	0.766	0.861	0.400
	Gemma-2	0.936	0.948	0.942	0.220
	Claude-2	0.785	0.805	0.795	0.210
	Random classifier	0.775	0.497	0.604	0.506
Protein related	GPT-3.5	0.500	0.897	0.642	0.480
	GPT-4	0.875	0.483	0.622	0.840
	Deepseek-R1-Distill	0.857	0.207	0.333	0.930
	Qwen-2.5	0.778	0.483	0.596	0.820
	Phi-4	1.000	0.069	0.129	0.980
	Llama-3.1	1.000	0.069	0.129	0.980
	Gemma-2	0.760	0.655	0.704	0.750

	Claude-2	0.303	0.931	0.457	0.110
	Random classifier	0.287	0.482	0.359	0.513

**Diabetes Mellitus**					
**single-prompt**	**Model**	**Precision**	**Recall**	**F1**	**Workload reduction**
Species	GPT-3.5	0.975	0.823	0.893	0.190
	GPT-4	0.989	0.917	0.951	0.110
	Deepseek-R1-Distill	0.979	0.958	0.968	0.060
	Qwen-2.5	1.000	0.990	0.995	0.050
	Phi-4	1.000	0.865	0.928	0.170
	Llama-3.1	0.979	0.990	0.984	0.030
	Gemma-2	0.969	0.990	0.979	0.020
	Claude-2	0.967	0.917	0.941	0.090
	Random classifier	0.957	0.494	0.651	0.504
Research type	GPT-3.5	0.449	1.000	0.620	0.110
	GPT-4	0.810	0.850	0.829	0.580
	Deepseek-R1-Distill	0.578	0.925	0.711	0.360
	Qwen-2.5	0.833	0.875	0.853	0.580
	Phi-4	0.833	0.750	0.789	0.640
	Llama-3.1	0.825	0.825	0.825	0.600
	Gemma-2	0.696	0.975	0.812	0.440
	Claude-2	0.484	0.775	0.596	0.360
	Random classifier	0.393	0.497	0.438	0.494
Disease_dm	GPT-3.5	0.837	0.554	0.667	0.510
	GPT-4	0.919	0.770	0.838	0.380
	Deepseek-R1-Distill	0.970	0.432	0.598	0.670
	Qwen-2.5	0.932	0.743	0.827	0.410
	Phi-4	0.929	0.527	0.673	0.580
	Llama-3.1	0.947	0.486	0.642	0.620
	Gemma-2	0.900	0.851	0.875	0.300
	Claude-2	0.766	0.797	0.781	0.230
	Random classifier	0.739	0.499	0.594	0.501
Disease_p	GPT-3.5	0.930	0.663	0.774	0.430
	GPT-4	0.937	0.925	0.931	0.210
	Deepseek-R1-Distill	0.953	0.762	0.847	0.360
	Qwen-2.5	0.961	0.925	0.943	0.230
	Phi-4	0.986	0.863	0.920	0.300
	Llama-3.1	0.958	0.863	0.908	0.280
	Gemma-2	0.951	0.963	0.957	0.190
	Claude-2	0.829	0.787	0.807	0.240
	Random classifier	0.801	0.494	0.609	0.506

**Sarcopenia**					
**single-prompt**	**Model**	**Precision**	**Recall**	**F1**	**Workload reduction**
Species	GPT-3.5	0.939	0.837	0.885	0.180
	GPT-4	0.987	0.826	0.899	0.230
	Deepseek-R1-Distill	0.989	0.989	0.989	0.080
	Qwen-2.5	0.986	0.793	0.879	0.260
	Phi-4	0.985	0.696	0.816	0.350

	Llama-3.1	1.000	0.935	0.966	0.140
	Gemma-2	0.968	0.989	0.978	0.060
	Claude-2	0.906	0.837	0.870	0.150
	Random classifier	0.920	0.496	0.643	0.504
Disease	GPT-3.5	0.733	0.904	0.810	0.100
	GPT-4	0.742	0.945	0.831	0.070
	Deepseek-R1-Distill	0.814	0.781	0.797	0.300
	Qwen-2.5	0.762	0.836	0.797	0.200
	Phi-4	0.778	0.863	0.818	0.190
	Llama-3.1	0.747	0.808	0.776	0.210
	Gemma-2	0.734	0.945	0.826	0.060
	Claude-2	0.714	0.753	0.733	0.230
	Random classifier	0.734	0.509	0.600	0.494
Control	GPT-3.5	0.704	0.838	0.765	0.190
	GPT-4	0.615	0.235	0.340	0.740
	Deepseek-R1-Distill	0.760	0.279	0.408	0.750
	Qwen-2.5	0.684	0.191	0.299	0.810
	Phi-4	NaN	0.000	NaN	1.000
	Llama-3.1	0.750	0.044	0.083	0.960
	Gemma-2	0.696	0.235	0.351	0.770
	Claude-2	0.677	0.618	0.646	0.380
	Random classifier	0.678	0.497	0.572	0.501
Research type	GPT-3.5	0.852	1.000	0.920	0.390
	GPT-4	0.977	0.827	0.896	0.560
	Deepseek-R1-Distill	0.943	0.962	0.952	0.470
	Qwen-2.5	1.000	0.827	0.905	0.570
	Phi-4	0.956	0.827	0.887	0.550
	Llama-3.1	0.977	0.827	0.896	0.560
	Gemma-2	0.979	0.904	0.940	0.520
	Claude-2	0.612	1.000	0.759	0.150
	Random classifier	0.517	0.492	0.502	0.506
Outcome	GPT-3.5	0.490	0.774	0.600	0.510
	GPT-4	0.793	0.742	0.767	0.710
	Deepseek-R1-Distill	0.633	0.613	0.623	0.700
	Qwen-2.5	0.703	0.839	0.765	0.630
	Phi-4	0.957	0.710	0.815	0.770
	Llama-3.1	0.875	0.677	0.763	0.760
	Gemma-2	0.617	0.935	0.743	0.530
	Claude-2	0.353	0.968	0.517	0.150
	Random classifier	0.317	0.498	0.386	0.513

Supplementary Table 2 Performance of three prompt strategies with the best combination and the full combination

Glioma		Best Combination				Full Combination			
**Model**	**Prompt strategy**	**Precision**	**Recall**	**F1**	**Work-reduced**	**Precision**	**Recall**	**F1**	**Work-reduced**
GPT-3.5	Prompt strategy 1	0.257	0.913	0.401	0.310	0.296	0.830	0.436	0.456
	Prompt strategy 2	0.246	0.932	0.390	0.265	0.471	0.772	0.585	0.678
	Prompt strategy 3	0.217	0.981	0.356	0.122	0.358	0.852	0.504	0.538
Random classifier		0.194	0.499	0.280	0.502	0.194	0.499	0.280	0.502
GPT-4	Prompt strategy 1	0.263	1.000	0.417	0.620	0.400	0.800	0.533	0.800
	Prompt strategy 2	0.119	1.000	0.213	0.160	0.159	0.700	0.259	0.560
	Prompt strategy 3	0.125	1.000	0.222	0.200	0.233	0.700	0.350	0.700
Deepseek-R1-Distill	Prompt strategy 1	0.125	0.600	0.207	0.520	0.000	0.000	NaN*	0.910
	Prompt strategy 2	0.143	1.000	0.250	0.300	0.081	0.300	0.128	0.630
	Prompt strategy 3	0.148	0.900	0.254	0.390	0.143	0.400	0.211	0.720
Qwen-2.5	Prompt strategy 1	0.157	0.800	0.262	0.490	0.318	0.700	0.437	0.780
	Prompt strategy 2	0.157	0.800	0.262	0.490	0.176	0.600	0.272	0.660
	Prompt strategy 3	0.152	0.700	0.250	0.540	0.217	0.500	0.303	0.770
Phi-4	Prompt strategy 1	0.118	1.000	0.211	0.150	0.462	0.600	0.522	0.870
	Prompt strategy 2	0.115	1.000	0.206	0.130	0.250	0.800	0.381	0.680
	Prompt strategy 3	0.130	1.000	0.230	0.230	0.500	0.700	0.583	0.860
Llama-3.1	Prompt strategy 1	0.185	1.000	0.312	0.460	0.556	0.500	0.527	0.910
	Prompt strategy 2	0.114	1.000	0.205	0.120	0.160	0.800	0.267	0.500
	Prompt strategy 3	0.125	1.000	0.222	0.200	0.200	0.800	0.320	0.600
Gemma-2	Prompt strategy 1	0.145	1.000	0.253	0.310	0.200	0.900	0.327	0.550
	Prompt strategy 2	0.111	1.000	0.200	0.100	0.195	0.800	0.314	0.590
	Prompt strategy 3	0.119	1.000	0.213	0.160	0.184	0.900	0.306	0.510
Claude-2	Prompt strategy 1	0.120	1.000	0.214	0.170	0.133	0.800	0.228	0.400
	Prompt strategy 2	0.068	0.500	0.120	0.270	0.099	0.800	0.176	0.190
	Prompt strategy 3	0.105	1.000	0.190	0.050	0.103	1.000	0.187	0.030
Random classifier		0.100	0.495	0.166	0.504	0.100	0.495	0.166	0.504

Inflammatory Bowel Diseases		Best Combination				Full Combination			
**Model**	**Prompt strategy**	**Precision**	**Recall**	**F1**	**Work-reduced**	**Precision**	**Recall**	**F1**	**Work-reduced**
GPT-3.5	Prompt strategy 1	0.042	0.978	0.081	0.188	0.047	0.444	0.086	0.670
	Prompt strategy 2	0.045	0.956	0.086	0.252	0.180	0.533	0.270	0.896
	Prompt strategy 3	0.044	0.978	0.084	0.212	0.084	0.778	0.152	0.675
Random classifier		0.036	0.514	0.067	0.502	0.036	0.514	0.067	0.502
GPT-4	Prompt strategy 1	0.152	1.000	0.263	0.340	0.500	0.200	0.286	0.960
	Prompt strategy 2	0.136	0.900	0.237	0.340	1.000	0.200	0.333	0.980
	Prompt strategy 3	0.154	1.000	0.267	0.350	0.500	0.100	0.167	0.980
Deepseek-R1-Distill	Prompt strategy 1	0.171	0.600	0.266	0.650	0.000	0.000	NaN*	0.960
	Prompt strategy 2	0.214	0.900	0.346	0.580	0.000	0.000	NaN*	0.890
	Prompt strategy 3	0.184	0.700	0.291	0.620	0.080	0.200	0.114	0.750
Qwen-2.5	Prompt strategy 1	0.300	0.600	0.400	0.800	0.500	0.100	0.167	0.980
	Prompt strategy 2	0.261	0.600	0.364	0.770	0.333	0.200	0.250	0.940
	Prompt strategy 3	0.455	0.500	0.476	0.890	1.000	0.100	0.182	0.990

Phi-4	Prompt strategy 1	0.385	0.500	0.435	0.870	0.000	0.000	NaN*	0.990
	Prompt strategy 2	0.308	0.800	0.445	0.740	0.167	0.100	0.125	0.940
	Prompt strategy 3	0.500	0.600	0.545	0.880	NaN**	0.000	NaN*	1.000
Llama-3.1	Prompt strategy 1	0.292	0.700	0.412	0.760	0.000	0.000	NaN*	0.990
	Prompt strategy 2	0.189	0.700	0.298	0.630	0.412	0.700	0.519	0.830
	Prompt strategy 3	0.214	0.900	0.346	0.580	0.304	0.700	0.424	0.770
Gemma-2	Prompt strategy 1	0.211	0.800	0.334	0.620	0.385	0.500	0.435	0.870
	Prompt strategy 2	0.170	0.800	0.280	0.530	0.357	0.500	0.417	0.860
	Prompt strategy 3	0.195	0.800	0.314	0.590	0.292	0.700	0.412	0.760
Claude-2	Prompt strategy 1	0.111	0.800	0.195	0.280	0.111	0.800	0.195	0.280
	Prompt strategy 2	0.108	1.000	0.195	0.070	0.108	1.000	0.195	0.070
	Prompt strategy 3	0.100	1.000	0.182	0.000	0.100	1.000	0.182	0.000
Random classifier		0.098	0.485	0.163	0.504	0.098	0.485	0.163	0.504

**Diabetes Mellitus**		**Best Combination**				**Full Combination**			
**Model**	**Prompt strategy**	**Precision**	**Recall**	**F1**	**Work-reduced**	**Precision**	**Recall**	**F1**	**Work-reduced**
GPT-3.5	Prompt strategy 1	0.173	0.823	0.287	0.434	0.165	0.379	0.230	0.726
	Prompt strategy 2	0.171	0.806	0.282	0.436	0.520	0.106	0.176	0.976
	Prompt strategy 3	0.133	0.782	0.227	0.295	0.150	0.847	0.255	0.322
Random classifier		0.118	0.492	0.190	0.502	0.118	0.492	0.190	0.502
GPT-4	Prompt strategy 1	0.125	1.000	0.222	0.280	0.263	0.556	0.357	0.810
	Prompt strategy 2	0.119	0.889	0.211	0.330	0.222	0.444	0.296	0.820
	Prompt strategy 3	0.130	0.778	0.222	0.460	0.143	0.667	0.235	0.580
Deepseek-R1-Distill	Prompt strategy 1	0.127	0.889	0.222	0.370	0.091	0.111	0.100	0.890
	Prompt strategy 2	0.140	0.889	0.242	0.430	0.175	0.778	0.286	0.600
	Prompt strategy 3	0.106	0.778	0.187	0.340	0.122	0.556	0.200	0.590
Qwen-2.5	Prompt strategy 1	0.167	0.778	0.275	0.580	0.278	0.556	0.371	0.820
	Prompt strategy 2	0.179	0.778	0.291	0.610	0.140	0.667	0.231	0.570
	Prompt strategy 3	0.123	0.889	0.216	0.350	0.333	0.444	0.381	0.880
Phi-4	Prompt strategy 1	0.129	0.889	0.225	0.380	0.250	0.333	0.286	0.880
	Prompt strategy 2	0.129	1.000	0.229	0.300	0.179	0.556	0.271	0.720
	Prompt strategy 3	0.125	0.556	0.204	0.600	0.135	0.778	0.230	0.480
Llama-3.1	Prompt strategy 1	0.175	0.778	0.286	0.600	0.273	0.333	0.300	0.890
	Prompt strategy 2	0.119	0.778	0.206	0.410	0.140	0.889	0.242	0.430
	Prompt strategy 3	0.127	0.778	0.218	0.450	0.222	0.444	0.296	0.820
Gemma-2	Prompt strategy 1	0.167	0.889	0.281	0.520	0.156	0.556	0.244	0.680
	Prompt strategy 2	0.145	0.889	0.249	0.450	0.138	1.000	0.243	0.350
	Prompt strategy 3	0.121	0.889	0.213	0.340	0.128	0.667	0.215	0.530
Claude-2	Prompt strategy 1	0.104	0.889	0.186	0.230	0.130	0.778	0.223	0.460
	Prompt strategy 2	0.092	0.889	0.167	0.130	0.082	0.889	0.150	0.020
	Prompt strategy 3	0.093	1.000	0.170	0.030	0.086	0.889	0.157	0.070
Random classifier		0.089	0.491	0.151	0.504	0.089	0.491	0.151	0.504

**Sarcopenia**		**Best Combination**				**Full Combination**			
**Model**	**Prompt strategy**	**Precision**	**Recall**	**F1**	**Work-reduced**	**Precision**	**Recall**	**F1**	**Work-reduced**
GPT-3.5	Prompt strategy 1	0.158	0.951	0.271	0.433	0.166	0.418	0.237	0.762
	Prompt strategy 2	0.166	0.975	0.284	0.445	0.286	0.148	0.195	0.951

	Prompt strategy 3	0.136	0.967	0.238	0.327	0.208	0.902	0.337	0.590
Random classifier		0.093	0.493	0.157	0.501	0.093	0.493	0.157	0.501
GPT-4	Prompt strategy 1	0.250	0.900	0.391	0.640	0.333	0.100	0.154	0.970
	Prompt strategy 2	0.250	0.900	0.391	0.640	0.500	0.600	0.545	0.880
	Prompt strategy 3	0.243	0.900	0.383	0.630	0.500	0.500	0.500	0.900
Deepseek-R1-Distill	Prompt strategy 1	0.191	0.900	0.315	0.530	0.000	0.000	NaN*	0.980
	Prompt strategy 2	0.175	1.000	0.298	0.430	0.179	0.700	0.285	0.610
	Prompt strategy 3	0.172	1.000	0.294	0.420	0.170	0.800	0.280	0.530
Qwen-2.5	Prompt strategy 1	0.278	1.000	0.435	0.640	0.000	0.000	NaN*	0.990
	Prompt strategy 2	0.364	0.800	0.500	0.780	0.304	0.700	0.424	0.770
	Prompt strategy 3	0.333	0.800	0.470	0.760	0.636	0.700	0.666	0.890
Phi-4	Prompt strategy 1	0.175	1.000	0.298	0.430	NaN**	0.000	NaN*	1.000
	Prompt strategy 2	0.125	1.000	0.222	0.200	0.412	0.700	0.519	0.830
	Prompt strategy 3	0.180	0.900	0.300	0.500	0.556	0.500	0.527	0.910
Llama-3.1	Prompt strategy 1	0.137	1.000	0.241	0.270	0.000	0.000	NaN*	0.970
	Prompt strategy 2	0.119	1.000	0.213	0.160	0.219	0.700	0.334	0.680
	Prompt strategy 3	0.116	1.000	0.208	0.140	0.350	0.700	0.467	0.800
Gemma-2	Prompt strategy 1	0.217	1.000	0.357	0.540	0.250	0.100	0.143	0.960
	Prompt strategy 2	0.120	1.000	0.214	0.170	0.219	0.700	0.334	0.680
	Prompt strategy 3	0.141	1.000	0.247	0.290	0.214	0.900	0.346	0.580
Claude-2	Prompt strategy 1	0.141	1.000	0.247	0.290	0.138	0.800	0.235	0.420
	Prompt strategy 2	0.071	0.600	0.127	0.150	0.063	0.500	0.112	0.210
	Prompt strategy 3	0.100	1.000	0.182	0.000	0.100	1.000	0.182	0.000
Random classifier		0.099	0.488	0.164	0.504	0.099	0.488	0.164	0.504

* Precision+Recall = 0; ** TP+FP = 0; The background red color is scaled in each column respectively.

Supplementary Files

Supplementary File 1 The content of three prompt strategies using full combination

Meta analysis: Glioma

Prompt with single criterion (single-prompt) / Prompt strategy 1 (Full combination)

Species

I want you to act as a helpful assistant. I will give you title and abstract of a publication and you will reply whether it meets our criteria or not. I want you to only reply with yes, no, or not sure, and followed with reasons. The criteria is: studies that use human as primary research subject.

Disease

I want you to act as a helpful assistant. I will give you title and abstract of a publication and you will reply whether it meets our criteria or not. I want you to only reply with yes, no, or not sure, and followed with reasons. The criteria is: studies that involve patients with glioma, glioblastoma, astrocytoma, oligodendroglioma.

Treatment

I want you to act as a helpful assistant. I will give you title and abstract of a publication and you will reply whether it meets our criteria or not. I want you to only reply with yes, no, or not sure, and followed with reasons. The criteria is: The title and abstract must explicitly mention that the study involves treatment of patients with one or more of the following immunotherapies: Immune Checkpoint Inhibitors (e.g. anti-PD-1, anti-PD-L1, anti-CTLA-4), Peptide Vaccination, Dendritic cell Vaccination, Adoptive Transfer of Effector Lymphocytes (e.g. CAR-T cell therapy, TCR-engineered T cells), Chimeric Antigen Receptor (CAR) T-cell therapy, or Oncolytic Viral Vectors therapy.

Research type

I want you to act as a helpful assistant. I will give you title and abstract of a publication and you will reply whether it meets our criteria or not. I want you to only reply with yes, no, or not sure, and followed with reasons. The criteria is: studies that was original research instead of reviews, protocol, or case report with less than five patients.

Chain of thought prompt (Prompt strategy 2; Full combination)

I want you to act as a helpful assistant. I will give you title and abstract of a publication and I want you to perform the following actions:

1. you will judge whether the publication meets the first criteria or not. The first criteria is: studies that use human as primary research subject.

2. you will judge whether the publication meets the second criteria or not. The second criteria is: studies that involve patients with glioma, glioblastoma, astrocytoma, oligodendroglioma.

3. you will judge whether the publication meets the third criteria or not. The third criteria is: The title and abstract must explicitly mention that the study involves treatment of patients with one or more of the following immunotherapies: Immune Checkpoint Inhibitors (e.g. anti-PD-1, anti-PD-L1, anti-CTLA-4), Peptide Vaccination, Dendritic cell Vaccination, Adoptive Transfer of Effector Lymphocytes (e.g. CAR-T cell therapy, TCR-engineered T cells), Chimeric Antigen Receptor (CAR) T-cell therapy, or Oncolytic Viral Vectors therapy.

4. you will judge whether the publication meets the fourth criteria or not. The fourth criteria is: studies that was original research instead of reviews, protocol, or case report with less than five patients.

5. you will summary the judgements from action 1 to 4. If the publication doesn't meet one or more of these criteria, you will answer 'No', otherwise, you will answer 'Yes'. Note that 'unclear' is treated as 'Yes'.

6.output a json object that contains the following keys: 'answer of action 1', 'answer of action 2', 'answer of action 3', 'answer of action 4', 'answer of action 5'.

Instruction prompt (Prompt strategy 3; Full combination)

I want you to act as a helpful assistant. I will give you title and abstract of a publication and you will reply whether it meets all

these following criteria or not: (1) studies that use human as primary research subject; (2) studies that involve patients with glioma, glioblastoma, astrocytoma, oligodendroglioma; (3) The title and abstract must explicitly mention that the study involves treatment of patients with one or more of the following immunotherapies: Immune Checkpoint Inhibitors (e.g. anti-PD-1, anti-PD-L1, anti-CTLA-4), Peptide Vaccination, Dendritic cell Vaccination, Adoptive Transfer of Effector Lymphocytes (e.g. CAR-T cell therapy, TCR-engineered T cells), Chimeric Antigen Receptor (CAR) T-cell therapy, or Oncolytic Viral Vectors therapy; (4) studies that was original research instead of reviews, protocol, or case report with less than five patients. Please answer in this way: If the publication doesn't meet one or more of these criteria, you will answer 'No', otherwise, you will answer 'Yes'. Note that 'unclear' is treated as 'Yes'.

Meta analysis: Inflammatory Bowel Diseases

Prompt with single criterion (single-prompt) / Prompt strategy 1 (Full combination)

Species

I want you to act as a helpful assistant. I will give you title and abstract of a publication and you will reply whether it meets our criteria or not. I want you to only reply with yes, no, or not sure, and followed with reasons. The criteria is: studies that only use human as research subject.

Disease

I want you to act as a helpful assistant. I will give you title and abstract of a publication and you will reply whether it meets our criteria or not. I want you to only reply with yes, no, or not sure, and followed with reasons. The criteria is: studies that involve patients with inflammatory bowel diseases, including Crohn's disease and ulcerative colitis.

Research type

I want you to act as a helpful assistant. I will give you title and abstract of a publication and you will reply whether it meets our criteria or not. I want you to only reply with yes, no, or not sure, and followed with reasons. The criteria is: studies that are prospective cohort study instead of case-contral study, cross-sectional study, retrospective cohort study, randomized controlled trial, review, or protocol.

Age

I want you to act as a helpful assistant. I will give you title and abstract of a publication and you will reply whether it meets our criteria or not. I want you to only reply with yes, no, or not sure, and followed with reasons. The criteria is: studies that involve adult patients (at least 18 years old).

Protein related

I want you to act as a helpful assistant. I will give you title and abstract of a publication and you will reply whether it meets our criteria or not. I want you to only reply with yes, no, or not sure, and followed with reasons. The criteria is: The title and abstract must mention that the study is related to the consumption of protein (e.g., total dairy, milk, meat, fish, poultry, process meat, and egg).

Chain of thought prompt (Prompt strategy 2; Full combination)

I want you to act as a helpful assistant. I will give you title and abstract of a publication and I want you to perform the following actions:

1. you will judge whether the publication meets the first criteria or not. The first criteria is: studies that only use human as research subject.

2. you will judge whether the publication meets the second criteria or not. The second criteria is: studies that involve patients with inflammatory bowel diseases, including Crohn's disease and ulcerative colitis.

3. you will judge whether the publication meets the third criteria or not. The third criteria is: studies that are prospective cohort study instead of case-contral study, cross-sectional study, retrospective cohort study, randomized controlled trial, review, or protocol.

4. you will judge whether the publication meets the fourth criteria or not. The fourth criteria is: studies that involve adult patients (at least 18 years old).

5. you will judge whether the publication meets the fifth criteria or not. The fifth criteria is: The title and abstract must mention that the study is related to the consumption of protein (e.g., total dairy, milk, meat, fish, poultry, process meat, and egg).

6. you will summary the judgements from action 1 to 5. If the publication doesn't meet one or more of these criteria, you will answer 'No', otherwise, you will answer 'Yes'. Note that 'unclear' is treated as 'Yes'.

7.output a json object that contains the following keys: 'answer of action 1', 'answer of action 2', 'answer of action 3', 'answer of action 4', 'answer of action 5', 'answer of action 6'.

Instruction prompt (Prompt strategy 3; Full combination)

I want you to act as a helpful assistant. I will give you title and abstract of a publication and you will reply whether it meets all these following criteria or not: (1) studies that only use human as research subject; (2) studies that involve patients with inflammatory bowel diseases, including Crohn's disease and ulcerative colitis; (3) studies that are prospective cohort study instead of case-contral study, cross-sectional study, retrospective cohort study, randomized controlled trial, review, or protocol; (4) studies that involve adult patients (at least 18 years old); (5) The title and abstract must mention that the study is related to the consumption of protein (e.g., total dairy, milk, meat, fish, poultry, process meat, and egg). Please answer in this way: If the publication doesn't meet one or more of these criteria, you will answer 'No', otherwise, you will answer 'Yes'. Note that 'unclear' is treated as 'Yes'.

Meta analysis: Diabetes Mellitus

Prompt with single criterion (single-prompt) / Prompt strategy 1 (Full combination)

Species

I want you to act as a helpful assistant. I will give you title and abstract of a publication and you will reply whether it meets our criteria or not. I want you to only reply with yes, no, or not sure, and followed with reasons. The criteria is: studies that only use human as research subject.

Research type

I want you to act as a helpful assistant. I will give you title and abstract of a publication and you will reply whether it meets our criteria or not. I want you to only reply with yes, no, or not sure, and followed with reasons. The criteria is: studies that are prospective or retrospective cohort study, case-control study. Of note, these research types doesn't meet the criteria: cross-sectional study, randomized controlled trial, review, protocol or others.

Disease_dm

I want you to act as a helpful assistant. I will give you title and abstract of a publication and you will reply whether it meets our criteria or not. I want you to only reply with yes, no, or not sure, and followed with reasons. The criteria is: studies that involve patients with diabetes mellitus.

Disease_p

I want you to act as a helpful assistant. I will give you title and abstract of a publication and you will reply whether it meets our criteria or not. I want you to only reply with yes, no, or not sure, and followed with reasons. The criteria is: studies that involve patients with parkinson's disease.

Chain of thought prompt (Prompt strategy 2; Full combination)

I want you to act as a helpful assistant. I will give you title and abstract of a publication and I want you to perform the following actions:

1. you will judge whether the publication meets the first criteria or not. The first criteria is: studies that only use human as research subject.

2. you will judge whether the publication meets the second criteria or not. The second criteria is: studies that are prospective or retrospective cohort study, case-control study. Of note, these research types doesn't meet the criteria: cross-sectional study, randomized controlled trial, review, protocol or others.

3. you will judge whether the publication meets the third criteria or not. The third criteria is: studies that involve patients with diabetes mellitus.

4. you will judge whether the publication meets the fourth criteria or not. The fourth criteria is: studies that involve patients with parkinson's disease.

5. you will summary the judgements from action 1 to 4. If the publication doesn't meet one or more of these criteria, you will answer 'No', otherwise, you will answer 'Yes'. Note that 'unclear' is treated as 'Yes'.

6.output a json object that contains the following keys: 'answer of action 1', 'answer of action 2', 'answer of action 3', 'answer of action 4', 'answer of action 5'.

Instruction prompt (Prompt strategy 3; Full combination)

I want you to act as a helpful assistant. I will give you title and abstract of a publication and you will reply whether it meets all these following criteria or not: (1)studies that only use human as research subject; (2) studies that are prospective or retrospective cohort study, case-control study. Of note, these research types doesn't meet the criteria: cross-sectional study, randomized controlled trial, review, protocol or others; (3)studies that involve patients with diabetes mellitus; (4) studies that involve patients with parkinson's disease. Please answer in this way: If the publication doesn't meet one or more of these criteria, you will answer 'No', otherwise, you will answer 'Yes'. Note that 'unclear' is treated as 'Yes'.

Meta analysis: Sarcopenia

Prompt with single criterion (single-prompt) / Prompt strategy 1 (Full combination)

Species

I want you to act as a helpful assistant. I will give you title and abstract of a publication and you will reply whether it meets our criteria or not. I want you to only reply with yes, no, or not sure, and followed with reasons. The criteria is: studies that only use human as research subject.

Disease

I want you to act as a helpful assistant. I will give you title and abstract of a publication and you will reply whether it meets our criteria or not. I want you to only reply with yes, no, or not sure, and followed with reasons. The criteria is: studies that involve patients with sarcopenia.

Control

I want you to act as a helpful assistant. I will give you title and abstract of a publication and you will reply whether it meets our criteria or not. I want you to only reply with yes, no, or not sure, and followed with reasons. The criteria is: studies that involve participants without sarcopenia.

Research type

I want you to act as a helpful assistant. I will give you title and abstract of a publication and you will reply whether it meets our criteria or not. I want you to only reply with yes, no, or not sure, and followed with reasons. The criteria is: studies that was original research instead of reviews, protocol, or others.

Outcome

I want you to act as a helpful assistant. I will give you title and abstract of a publication and you will reply whether it meets our criteria or not. I want you to only reply with yes, no, or not sure, and followed with reasons. The criteria is: The title and abstract must explicitly mention that the study reported health-related quality of life assessment.

Chain of thought prompt (Prompt strategy 2; Full combination)

I want you to act as a helpful assistant. I will give you title and abstract of a publication and I want you to perform the following actions:

1. you will judge whether the publication meets the first criteria or not. The first criteria is: studies that only use human as research subject.

2. you will judge whether the publication meets the second criteria or not. The second criteria is: studies that involve patients with sarcopenia.

3. you will judge whether the publication meets the third criteria or not. The third criteria is: studies that involve participants without sarcopenia.

4. you will judge whether the publication meets the fourth criteria or not. The fourth criteria is: studies that was original research instead of reviews, protocol, or others.

5. you will judge whether the publication meets the fifth criteria or not. The fifth criteria is: The title and abstract must explicitly mention that the study reported health-related quality of life assessment.

6. you will summary the judgements from action 1 to 5. If the publication doesn't meet one or more of these criteria, you will answer 'No', otherwise, you will answer 'Yes'. Note that 'unclear' is treated as 'Yes'.

7.output a json object that contains the following keys: 'answer of action 1', 'answer of action 2', 'answer of action 3', 'answer of action 4', 'answer of action 5', 'answer of action 6'.

Instruction prompt (Prompt strategy 3; Full combination)

I want you to act as a helpful assistant. I will give you title and abstract of a publication and you will reply whether it meets all

these following criteria or not: (1) studies that only use human as research subject; (2) studies that involve patients with sarcopenia; (3) studies that involve participants without sarcopenia; (4) studies that was original research instead of reviews, protocol, or others; (5) The title and abstract must explicitly mention that the study reported health-related quality of life assessment. Please answer in this way: If the publication doesn't meet one or more of these criteria, you will answer 'No', otherwise, you will answer 'Yes'. Note that 'unclear' is treated as 'Yes'.

Supplementary File 2 Validation datasets from four meta-analyses

See separate file.

Supplementary File 3 Supplementary Methods

Supplementary method of LARS pipeline

Selection of validation records from the inflammatory bowel diseases (IBD), diabetes mellitus (DM), and sarcopenia meta-analyses

Based on the detailed methods from these 3 published meta-analyses, we successfully repeated their literature research and classification of records. For these 3 validation datasets, all records were used for GPT-3.5 evaluation. However, only 100 randomly selected records were used for the GPT-4 evaluation, due to the limited funding. To do the randomization, each record was assigned a randomized number with the RAND function from EXCEL. The records with the top 100 randomized numbers were selected. The percentage of positive records (definition of positive/negative can be found in the Methods section of the manuscript) was checked after randomization. The percentages of positive records are around 10% for all three validation datasets (9 or 10 out of 100).

Selection of validation records from the glioma meta-analysis

Due to the large number of records (6020) identified from PubMed in the glioma meta-analysis, which was too much for evaluating ChatGPT, we opted to select a subset of these records. At first, we randomly selected 100 records for GPT-4 analysis (96 negative and 4 positive records). To make sure the glioma dataset has similar percentage of positive records as other validation datasets (around 10%), we randomly selected another 6 positive records to replace 6 randomly selected negative records in this dataset with 100 records. Additionally, we randomly selected 1000 negative records, together with these previously selected 96 negative records to test GPT-3.5. Also, all 264 positive records, which were identified from PubMed, were also included in the glioma validation dataset for GPT-3.5. In the end, a total of 1360 records were used for GPT-3.5 evaluation (**Table 2**).

Evaluation of response robustness of ChatGPT

Because of the nature of LLMs, the generated answer from LLMs varies each time, even with exactly identical input. We assessed the robustness of ChatGPT's answers using single-prompts before assessing the LARS-GPT pipeline. In other words, the robustness evaluation is a pre-assessment for ChatGPT. It's not a part of the LARS-GPT pipeline.

To evaluate the robustness of all three different answers ("Yes", "No", "Not sure"), we applied single-prompts on all records before testing the robustness. For each single-prompt, we randomly selected three records for each type of answer ("Yes", "No", "Not sure). Because of the overlap of randomly selected records, deduplication of selected records is conducted. In the end, a total of 74 records were used. And 10 repeated requests for each record were sent to ChatGPT for robustness assessment (see Fig below).

Then, for each single-prompt, we have:

$$\text{Robustness score} = \frac{\sum_{k=1}^{n} \max\left(P_k(Yes), P_k(No), P_k(Not\ sure)\right)}{n}$$

Where n is the number of selected records and P is the abbreviation of probability.

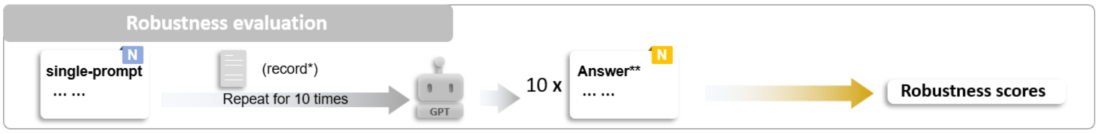

* Record: [Title: < *title of a record* >. Abstract: < *abstract of a record* >.]

** Answer: Yes / No / Not sure (judgement for single-prompt)

Statistical analysis

The random classifier was used as a baseline reference. This classifier randomly assigned an answer of "Yes" or "No" to each record, with 50% chance for each option. To obtain a stable baseline performance, the evaluation for the random classifier was iterated 100 times. The recall and workload reduction of random classifier were both around 0.5, which was set as a reference level.

Python was used to access ChatGPT and LM Studio. All statistical analyses were performed in R software (version 4.1). Upset plot

was drawn with "ComplexHeatmap" R package (version 2.10). Shapiro-Wilk test and Levene's test were applied to check the normality and the homogeneity of variance across groups, respectively. If a normality assumption was met, repeated measures ANOVA test ("rstatix" R package, version 0.7) was used to compare performance between groups. Otherwise, Kruskal–Wallis test would be used ("stats" R package, version 4.1) to estimate if all groups have the same distribution. If not, nonparametric multiple comparisons will be conducted with "nparcomp" R package (version 3.0). $P < 0.05$ was applied as indicator for statistical significance.
